# Lack of Critical Slowing Down Suggests that Financial Meltdowns Are Not Critical Transitions, yet Rising Variability Could Signal Systemic Risk

**DOI:** 10.1371/journal.pone.0144198

**Published:** 2016-01-13

**Authors:** Vishwesha Guttal, Srinivas Raghavendra, Nikunj Goel, Quentin Hoarau

**Affiliations:** 1 Centre for Ecological Sciences, Indian Institute of Science, Bengaluru, 560012, India; 2 J. E. Cairnes School of Business and Economics, National University of Ireland, Galway, Ireland; 3 Centre for Contemporary Studies, Indian Institute of Science, Bengaluru, 560012, India; 4 Ecole Normale Supérieure de Cachan, 94235 Cachan, France; Feng Chia University, TAIWAN

## Abstract

Complex systems inspired analysis suggests a hypothesis that financial meltdowns are abrupt critical transitions that occur when the system reaches a tipping point. Theoretical and empirical studies on climatic and ecological dynamical systems have shown that approach to tipping points is preceded by a generic phenomenon called critical slowing down, i.e. an increasingly slow response of the system to perturbations. Therefore, it has been suggested that critical slowing down may be used as an early warning signal of imminent critical transitions. Whether financial markets exhibit critical slowing down prior to meltdowns remains unclear. Here, our analysis reveals that three major US (Dow Jones Index, S&P 500 and NASDAQ) and two European markets (DAX and FTSE) did not exhibit critical slowing down prior to major financial crashes over the last century. However, all markets showed strong trends of rising variability, quantified by time series variance and spectral function at low frequencies, prior to crashes. These results suggest that financial crashes are not critical transitions that occur in the vicinity of a tipping point. Using a simple model, we argue that financial crashes are likely to be stochastic transitions which can occur even when the system is far away from the tipping point. Specifically, we show that a gradually increasing strength of stochastic perturbations may have caused to abrupt transitions in the financial markets. Broadly, our results highlight the importance of stochastically driven abrupt transitions in real world scenarios. Our study offers rising variability as a precursor of financial meltdowns albeit with a limitation that they may signal false alarms.

## Introduction

Financial markets can undergo catastrophic meltdowns and may endure a delayed recovery as witnessed in the major crashes of 1929 and 2008. Prevalence of such crashes in various markets around the world and their adverse impact on the global economy has reinforced the need for a more intensive inquiry from a variety of perspectives on the determinants of financial crashes. In addition, it is pertinent to ask whether there are any early warning signals (EWS) of impending crises. An emerging view of stock market crashes is that they could be driven by nonlinear feedbacks and stochasticities internal to the system. Mean-field macroeconomic models and microscopic agent based models that incorporate feedback between behaviors of investors and the state of the system show abrupt switches between bullish and bearish phases of markets. Furthermore, they capture empirically observed properties of variabilities in return rates, also called volatility [1–9].

Despite the success of these models in generating these *stylized facts* of financial markets, their utility as predictive tools has been limited because of challenges such as empirical estimation of model parameters corresponding to investor and/or market behaviors. Thus far, the development of early warning signals (EWS) of systemic risks in financial markets are largely based on statistical models [10–13]. For example, empirical observation of volatility prior to 1987 crash has led to devising statistical estimators of volatility as EWS of impending financial crises [10, 14–16]. More recently, system risk in financial systems has been shown to be preceded by increasing cross correlations or information dissipation in various financial sectors [17–22]. However, integrating theoretical approaches based on nonlinear dynamical systems with empirical and statistical methods to devise EWS of impending financial crises remain one of the open challenges.

Recent research has shown that nonlinear complex dynamical systems, such as climatic and ecological systems, may exhibit tipping points at which the system will abruptly shift from one state to another. Such transitions, also referred to as critical transitions, are qualitatively similar to financial meltdowns in exhibiting discontinuous state changes and delayed recovery to the original state [1, 23–25]. From a dynamical systems perspective, tipping points can be viewed as bifurcation points at which the stability of an equilibrium undergoes a qualitative change (see Methods A). Theory shows that the system considerably slows down in its response to perturbations as it approaches a bifurcation point [26, 27]. This phenomenon, known as ‘critical slowing down’, is expected to cause an increasing trend of autocorrelation which can be readily measured using time series data of the dynamical system [28, 29]. Furthermore, the system exhibits an increased variability and reddening of power-spectrum in its time series dynamics [30–32]. Therefore, it has been argued that these generic statistical indicators could be used as robust early warning signals of impending critical transitions [27, 29, 33–36]. These theoretical predictions have been empirically tested using data from past climatic shifts, and laboratory and field experiments in ecological systems ranging from microbial populations to lake ecosystems [28, 37–39]. In the context of these theoretical and empirical advances, it has been suggested that financial meltdowns are akin to abrupt critical transitions that occur near the tipping point of a system even for small perturbations [22, 27, 40].

**Fig 1:**
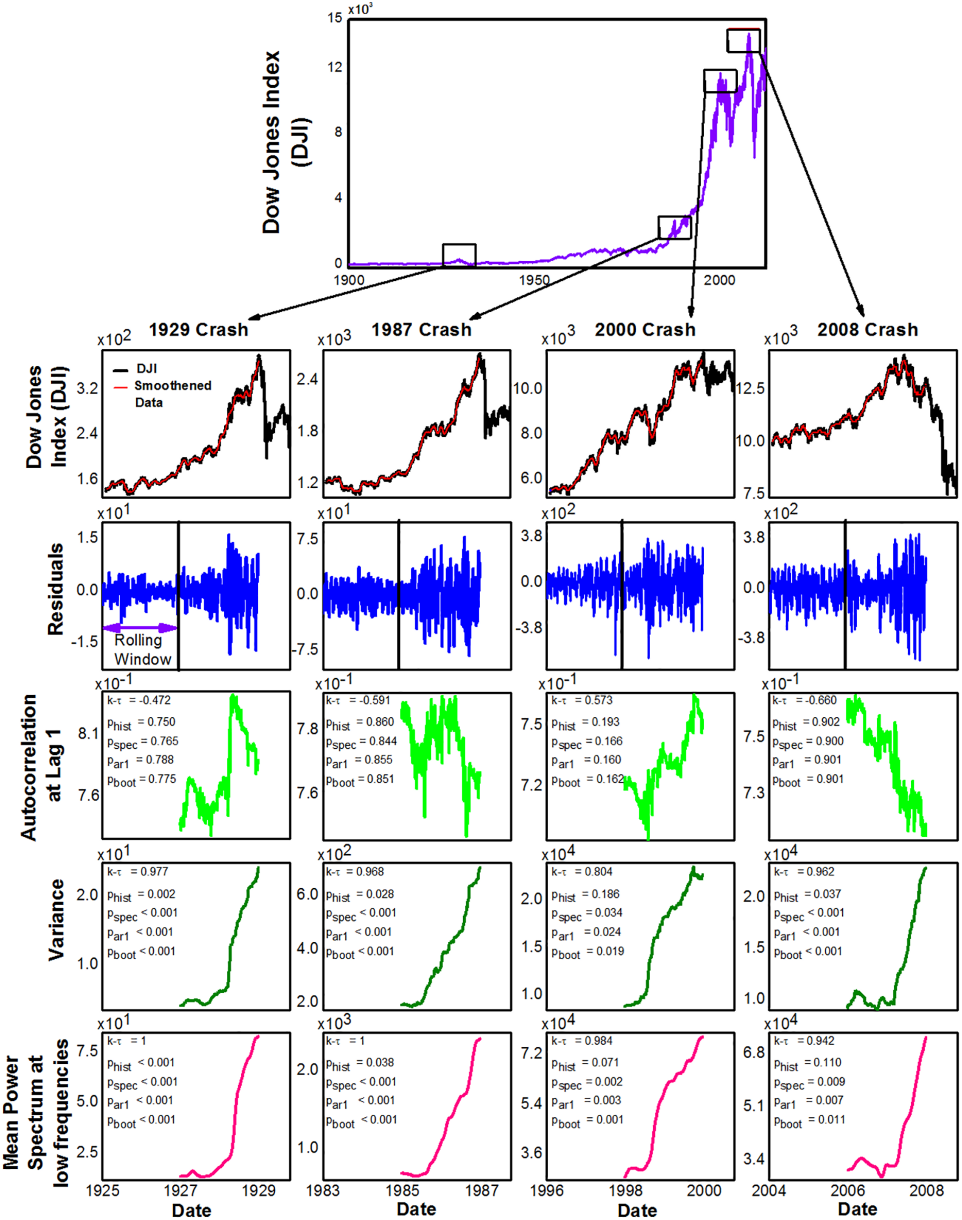
Early warning signals of major financial crashes of Dow Jones Index (DJI). The columns correspond to analysis of each crash. The solid vertical line together with the arrow in the residual plots, which were obtained after detrending the data, shows the length of the rolling window (*l*_*rw*_) over which all indicators are estimated. The symbol k-*τ* represents Kendall’s rank correlation coefficient estimated for each indicator for 250 days prior to the crash. The p-value denoted by *p*_*hist*_ quantifies how likely such trends are in years far away from the crash. The other three p-values quantify likelihood of such trends occurring by chance (Methods B). Parameters: *l*_*rw*_ = 500 days, *bw* = 25, *l*_*kw*_ = 250 days, *l*_*Kend*_ = 0 days.

**Fig 2:**
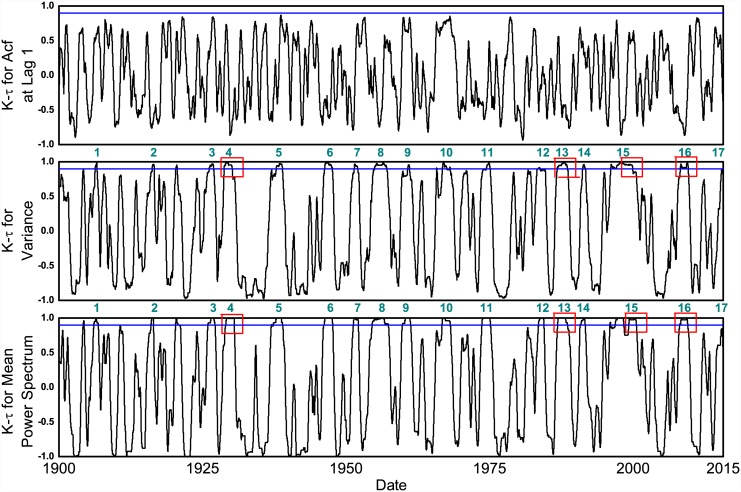
Strength of trends, as measured by Kendall-*τ*, in all three indicators for DJI as a function of time over the last century. The blue line is a threshold value of Kendall-*τ* (0.9) which is sufficiently large to conclude a strong increasing trend. Clearly, the trends for acf at lag 1 are weak and never exceed this threshold. For both variance and power spectrum, a total of sixteen events of crossing the threshold value were found and they are all listed in Table 1. Four of them indicated in red squares in the middle and the lower panel correspond to four major financial crashes, namely 1929, 1987, 2000 and 2008 whereas five others correspond to minor economic crises of last century. This figure also illustrates instances of strong trends of EWS that were not followed by any crash, suggesting that they were false alarms.

**Fig 3:**
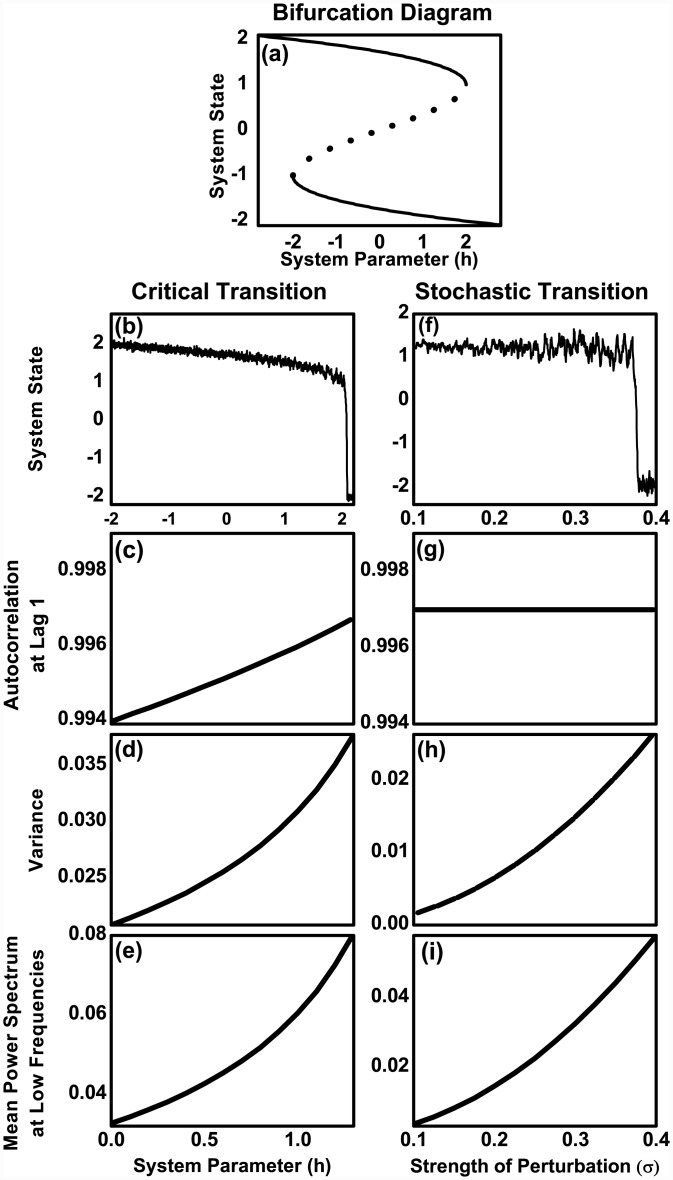
Early warning signals of critical transitions versus stochastic transitions. (A) The bifurcation diagram for the model $\dot{x}=-h+rx-x^{3}$, where *x* represents system state and *h* and *r* are system parameters (Methods A)). Solid lines represent stable equilibria and the dotted line represents unstable equilibria. In the left column, we show the dynamics and the early warning signals of the system, $\dot{x}=-h+rx-x^{3}+\sigma \eta \left(t\right)$, where *η*(*t*) is a Guassian white noise, i.e. 〈*η*(*t*)*η*(*t*′)〉 = *δ*(*t*−*t*′), as the system approaches critical point (the driver *h* → *h*_*c*_ = 2 with *r* = 3 and *σ* = 0.1). The right column shows abrupt transitions driven by increasing strength of perturbations (we increase *σ* with constant *r* = 3 and *h* = 0).

Motivated by these studies, we set out to ask whether financial crashes are indeed critical transitions and whether there are any EWS of impending economic crises. Here, we investigate these questions in the context of well known financial crashes in three major US stock markets and two European markets. More specifically, we conducted rigorous time series analysis to test whether financial markets exhibited critical slowing down prior to financial meltdowns. We found that all of the five major markets that we analysed showed no critical slowing down, hence challenging the earlier claim that financial crashes are critical transitions (Fig 1). However, we found consistent and strong trends of rising variability prior to crashes (Fig 1). Moreover, such trends were absent when the system was far away from a crash (Fig 2). Using a simple mathematical model (Methods A section), we argue that such characteristics of markets prior to crashes are better explained by stochastic transitions where a crash occurs even when the system is far away from the tipping point. We show that an increasing strength of stochastic perturbations can cause such transitions (Fig 3). These analyses suggest that robust occurrence of rising variability prior to crashes could be an early warning signal of financial crashes. We discuss implications of these results including limitations of EWS and comparison of rising variability to other indicators such as volatility in the Discussion section.

## Results

We have described two mathematical models, a simple mean-field model of abrupt transitions and a microeconomic agent-based model, that were used to make predictions about early warning signals of abrupt transitions in Methods A section. Detailed methodology of time series analysis is presented in Methods B section. To enable reproducibility of our results, we have deposited the computer codes of analysis along with data at the open source Github repository (https://github.com/tee-lab/indicators-financial-meltdowns). We describe the results below.

### Lack of critical slowing down but rising variability prior to crashes

We first analysed the daily closing data of Dow Jones Index (DJI) prior to four well known crashes of 1929, 1987, 2000, and 2008 (Fig 1). We detrended the data by removing long term trends in the chosen four year window using Gaussian kernel smoothing function. Our analysis of detrended time series (or residuals) showed that autocorrelation at lag-1 (henceforth, acf), a key measure of critical slowing down, showed either no or weak trends, as quantified by Kendall-*τ* rank correlation coefficient, prior to any of the crashes (see Methods B for details of time series analysis). In contrast, variance and the average of the power-spectrum at low frequencies both showed strong increasing trends prior to all of the crashes (Fig 1). To test if there was reddening of power-spectrum, we computed the average power spectrum at higher frequencies, which showed increasing trends that was comparable to increases in low frequency ranges; thus we found no evidence for reddening of power spectrum. These results were robust for a wide range of parameters, such as the size of the rolling window, the bandwidth of detrending, etc, chosen to analyse the data (Fig C3(b) in S1 File).

### Trends of indicators away from the major crashes

We then explored whether the trends we saw above were common even for randomly chosen periods of same length but far away from the crash. We found that, in years far away from the crash, the strong trends of increasing variance and power-spectrum were extremely rare (*p*_*hist*_ in Fig 1 represents the proportion of such events in the past). We identified all events exhibiting strong trends of variance or power spectrum (such that their Kendall-*τ* > 0.9) in the entire 115 year daily data of DJI. We found sixteen such events (Fig 2) five of which occurred prior to four major crashes. Four other events provided EWS in variance and power spectrum for relatively minor economic crises of the last century, arising from the panic of 1907, recession of 1937, oil shock of 1973 and Israeli oil crisis of 1983 (Table 1); the acf at lag-1 showed no strong trends prior to any of these events. The remaining seven events with strong trends in variance and power spectrum, of which five were in the period from 1945 to 1970, occurred with no known economic crises in their vicinity; we therefore conclude that they were false positives. Thus, our analyses showed that all important recorded stock market crises in DJI were preceded by EWS in variance and power spectrum at least three months in advance (i.e. no false negatives) but there were seven false alarms over the 115 year period of the analyzed data.

**Table 1:** Table of events of strong trends of early warning signals.

**Event number**	**Duration of high-Kendall in Power spectrum**	**Nearest recorded crash (start date & source)**	**EWS (time to crash) or False alarm**
1	17/08/1906 to 09/11/1906	Panic of 1907 (14/03/1907)	EWS (4 months)
2	26/07/1916 to 06/10/1916	none	False alarm
3	29/06/1926 to 05/05/1927	1929 Crash (24/10/1929)	EWS (3 years)
4	29/02/1929 to 14/10/1929	1929 Crash (24/10/1929)	EWS (9 months)
5	13/04/1937 to 23/02/1939	1937 recession (26/9/1937)	EWS (5 months)
6	30/07/1946 to 08/10/1947	none	False alarm
7	30/08/1951 to 15/05/1952	none	False alarm
8	26/11/1954 to 10/05/1957	none	False alarm
9	02/11/1959 to 14/04/1961	none	False alarm
10	26/09/1966 to 06/03/1968	none	False alarm
11	06/09/1973 to 09/03/1975	Oil shock (2/11/1973)	EWS (3 months)
12	15/07/1983 to 27/06/1984	Israel oil crisis (10/10/1983)	EWS (3 months)
13	12/11/1986 to 04/08/1988	1987 Crash (19/10/1987)	EWS (11 months)
14	12/10/1990 to 30/08/1991	none	False alarm
15	26/12/1995 to 09/08/2000	Dot-com crash (21/03/2000)	EWS (5 years)
16	15/02/2008 to 14/07/2009	2008 Crash (01/10/2008)	EWS (9 months)

**Table of events of strong trends**, as determined by Kendall-*τ* exceeding a threshold value of 0.9 in both variance and power spectrum (see caption for Fig 2). Event numbers in this table correspond to those identified in Fig 2. Note that there are several false alarms, as discussed in the text. In addition, there were persistent EWS for five years preceding 2000 crash. Whereas, in most cases of EWS the crashes occurred within an year or so of the signal. This indicates that occurrence of an EWS is not a predictive measure of when the crash will occur.

### Statistical significance of trends

To test whether observed trends occurred by chance alone, we employed three well developed null models of time series analysis using simulated data that maintains certain characteristics of the original time series (see Methods B for details) [28, 29]. These analyses suggested that the obtained trends of rising variability prior to crashes were unlikely to occur by chance alone. However, trends of acf of real data were comparable to those in simulated data, suggesting that our conclusions above are statistically significant. Finally, sensitivity analysis revealed that these results based on computing p-values were robust to changes in various parameters associated with time series analysis (Fig D in S1 File).

### Similar trends were found in S&P 500 and NASDAQ

To test whether these results are not specific to DJI alone, we analysed the daily data of S&P 500 and NASDAQ for three crashes, 1987, 2000 and 2008 (*Figs E and H in* S1 File). Here too, there was no critical slowing down prior to crashes. We found increasing trends of variance and power spectrum prior to crashes of 1987 and 2008, although they had lower statistical support in comparison to DJI (Figs F and I in S1 File). However, the trends of variance and power spectrum prior to the 2000 crash, also called the dot-com bubble, were much stronger for the NASDAQ than for both DJI and S&P 500. This is consistent with the fact that the 2000 crash was primarily influenced by the information technology sector that directly affects NASDAQ. Analysis of high frequency data (1 min) of DJI (2000 & 2008), S&P 500 (1987, 2000 & 2008) and NASDAQ (2008) yielded qualitatively similar results (not shown).

### Similar trends were found in German and UK markets

It is well known that some of the recent financial crises were global in nature, affecting other major economies of the world. Therefore, it is natural to ask whether same trends hold true in other major stock markets. We chose two major European markets: the DAX (Deutscher Aktienindex or German stock index), a stock market consisting of 30 major German companies trading in Frankfurt Stock Exchange and the FTSE (the Financial Times Stock Exchange 100 Index) which is a major stock market listed on the London Stock Exchange. We analysed these market data prior to crashes of 2000 and 2008. In both of these markets, there was no critical slowing down prior to either of the crashes (Figs L and M in S1 File). In addition, like in other markets, we found strong trends of increasing variance and power spectrum at low frequencies. In other words, the trends of indicators of German and UK stock markets prior to global financial crashes are qualitatively similar to those of the major US markets.

### Variance versus volatility

In our analysis above, we computed the variance of stock market time series based on the methods developed to detect early warning signals of abrupt transitions [29]; we first removed the low frequency fluctuations from the stock market time series and then computed the variance of the residual time series (Methods B). As we remarked in the introduction section, there is substantial research on measuring volatility of stock markets. Volatility too captures variability in the stock market time series, and more precisely the variability in return rates (see Text B and Fig N in S1 File). Although there are various definitions of volatility that slightly differ from each other, we employed the definition of Poon and Granger [41] where Volatility is equal to the variance (*σ*^2^) of stock returns

$${\hat{\sigma }}^{2}=\sum_{t=1}^{T}\frac{{\left(R_{t}-\bar{R}\right)}^{2}}{T-1}$$ (1)

where $\bar{R}$ is the mean of the returns. The return itself is calculated from the stock prices *p*_*t*_ as

$$R_{t}=\log\left[\frac{p_{t+1}}{p_{t}}\right]\approx \frac{p_{t+1}-p_{t}}{p_{t}}$$ (2)

The above Eq (2) is a reasonable approximation in the case of high frequency data. Despite apparent similarity between variance and volatility, the results of variance of residuals and volatility of return rates are not always qualitatively similar (Fig N in S1 File). For example, we found that volatility did not exhibit consistent strong increasing trends prior to 1987 crash for NASDAQ data, and 2000 crash for both DJI and S&P 500.

### Critical transitions or Stochastic transitions

Thus far, our analysis of various stock markets crashes provides evidence for increasing trends in variance and power-spectrum at all frequencies prior to transitions with no (or statistically weak) trends in acf. These results present an anomaly to the theory of critical transitions where critical slowing down is expected to occur en route to tipping point. This led us to investigate the nature of these transitions. Abrupt transitions can also occur far away from the tipping point on two accounts: (a) when the strength of stochastic perturbations are large [31, 42], (b) when the strength of stochastic perturbations increase gradually as the system evolves (Methods A). We refer to them as stochastic transitions. The former route to stochastic transition where the system is hit by a large stochastic shock may not exhibit EWS. We substantiate this point using two well known economic theoretic models, a stochastic macroeconomic model that exhibits bistable dynamics and a microeconomic heterogeneous agent based model where investors’ behaviour is probabilistic [43, 44]. In both of these models, we found that the first type of stochastic transitions typically do not exhibit any EWS (Text A and Figs A and B in S1 File).

We investigated the second type of stochastic transition by gradually increasing the strength of perturbations in a simple and a general model of abrupt transitions with stochasticity. We found that this type of stochastic transitions is preceded by a rising variance and power-spectrum at all frequencies (i.e., no reddening of power spectrum) with no trends in autocorrelation at lag-1 (Methods A and Fig 3). Since the analysis of stock markets too showed rising variance with no trends in autocorrelation at lag-1, we infer that financial crashes may have been driven by an increasing strength of stochastic fluctuations.

### Recent trends of indicators

**Fig 4:**
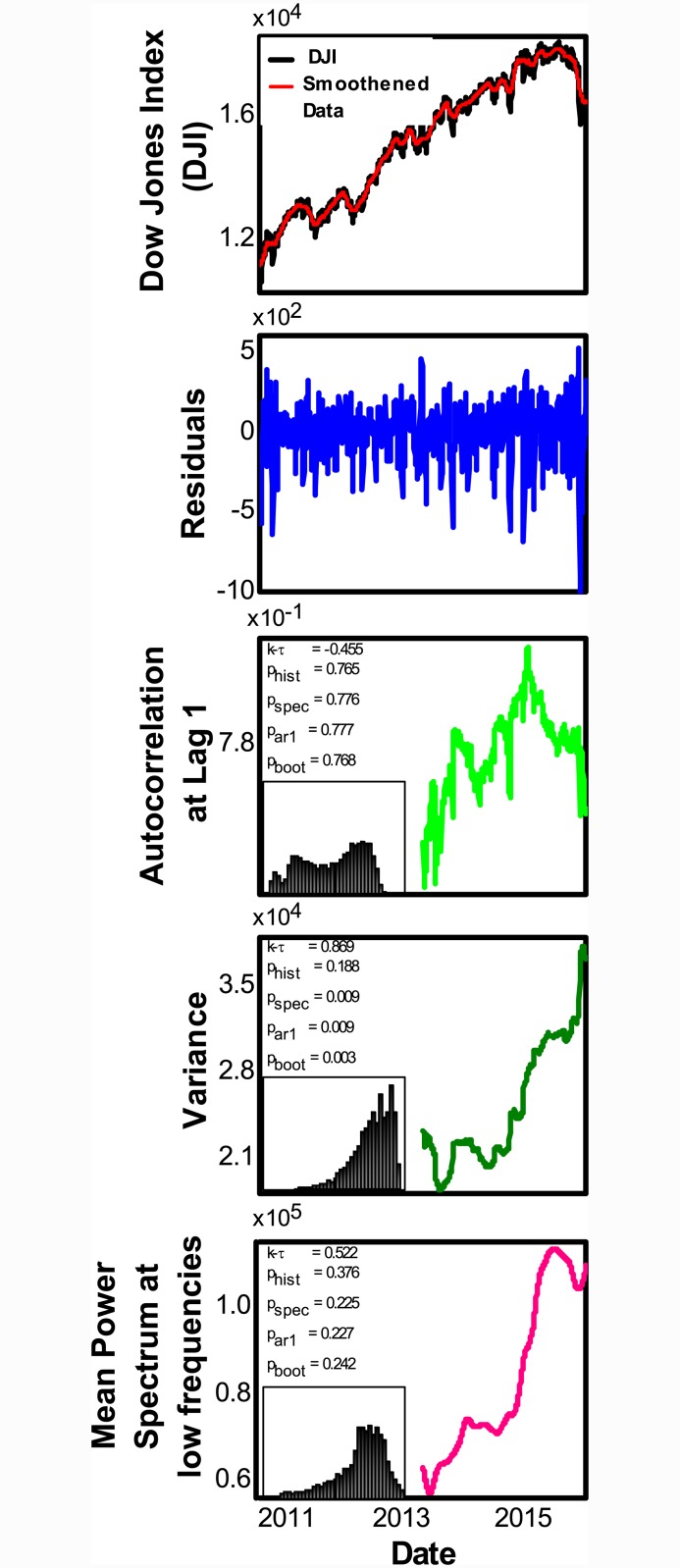
Lack of significant increasing trends of indicators for DJI data for the duration 17/09/2011 to 16/09/2015. Parameters used to analyze data are same as in Fig 1. The insets show a distribution of Kendall-*τ* for the corresponding indicator obtained by a parameter scan of over 1.25 million combinations. Lack of consistent value in Kendall−*τ* and relatively high p-values (> 0.1) for all EWS suggest that there are no clear trends. For the inset: x-axis scale is from −1 to 1 and the y-axis is from 0 to 0.11 (See Methods B).

Based on our investigation of four major US stock market crashes, which suggests that increasing variance and power-spectrum could be reliable precursors of financial crashes, we analysed the five stock market data, post 2008 crash, from Sept 2011 to Sept 2015. We did not find strong and statistically significant trends for variability in DJI (Fig 4) S&P500 and FTSE (Figs G and M(b) in S1 File). However, recent trends of NASDAQ (Fig J in S1 File) and German indices (Fig M(a) in S1 File) show strong increases in variability. Put together, these markets currently show mixed early warning signals, potentially reflecting the global economic stress induced by recent Chinese financial crisis. Based on our analysis of previous crashes, we argue that a sustenance of rising variability in markets over next few months could indicate impending financial crises in these markets.

## Discussion

Our analyses of three major financial markets of the US and two of Europe show that they did not exhibit critical slowing down prior to financial crashes. However, financial crashes were preceded by strong and consistent trends of rising variability prior to all of the crashes. Using a simple model, we argue that these features are not consistent with critical transitions, which are abrupt transitions that occur even for small perturbations near a tipping point. Rather, these are better explained by abrupt transitions that are driven by increasing strength of stochastic perturbations. Furthermore, our results suggest that rising variability could be employed as early warning signals of imminent financial crises, albeit with some limitations. We discuss various implications of our results as well as limitations of employing rising variability as an early warning signal below.

### Stochastic vs critical transitions

Let us look at our results context of two broad frameworks that are widely employed to explain financial crashes. Within the framework of efficient market hypothesis, crashes are a consequence of the system being subjected to unexpected large external shocks and thus there could be no early warning signals. An alternative approach, inspired by complex systems research, is that crashes happen even for small perturbations due to internal dynamics when the system is driven close to a tipping point. Abrupt changes that occur near a tipping point are called critical transitions. Theories predict that such transitions are preceded by critical slowing down. In contrast to both of these perspectives, our analyses suggests that financial meltdowns are likely driven by gradually increasing strength of stochastic perturbations that results in an abrupt transition even when the system is far away from the tipping point.

Much of the previous work on abrupt transitions in complex dynamical systems, such as climatic and ecological systems, have focused on critical transitions [27, 28, 37–39]. Complex systems across scales are strongly influenced by external perturbations. However, studies on stochastically driven abrupt transitions have received much less attention in the literature. Testing for the nature of stochasticity and nonlinearity in complex systems has been the focus of much research in various disciplines [45–48]. Our analysis based on the simpler low dimensional model with stochasticity as well as an heterogeneous agent based model does not inform us about the origin of these stochastic fluctuations, i.e., the whether the stochasticity is of exogenous or endogenous origin. It could potentially arise due to an increase in the temporal variability of a system parameter, for example due to the varying ability of agents in extracting signals from noise, or could be through interactions among agents. A future direction of work would be to develop agent based models that capture the empirical characteristics of lack of critical slowing down with rising variability prior to financial crashes.

### Early warning signals and their limitations

Our analyses suggest that financial crashes, despite their stochastic origin, could be preceded by strong increasing trends of variability, thus potentially offering early warning signals for future financial meltdowns. Early warning signals, however, are not without limitations. First, they are not predictive, i.e., they do not forecast when a crash might occur. For example, due to inherent stochastic nature of financial markets, we do not have a framework to convert recent strong trends of rising variability in various markets (Figs I and M(a) in S1 File) to forecast the next financial crisis. Furthermore, any system of early warning signals is inevitably prone to both false and failed alarms [9, 27]. Our analysis of lesser known bullish runs show that seven events of strong increase in variability were not followed by any financial meltdowns. However, there were no failed alarms, i.e., all known crashes were preceded by rising variability (Fig 2). Furthermore, we also found that incidences of false alarms could be increasing, as evidenced by increasing values of *p*_*hist*_ from 1929 crash to recent crashes. Therefore, translating these signals to policy actions requires a decision theoretic framework that enables the analysis of costs and benefits of potential alternative forecasts.

### Other indicators: Volatility, cross-correlations and liquidity

A commonly used measure of variability in the finance literature is volatility. A key difference between variance as we have computed and the measure of volatility is as follows. We computed variance of the residuals, which is obtained by removing longer time scale trends of the stock market time series. Volatility, on the other hand, is a measure of variance in the rate of return calculated from price of stocks without removing any time trends in the data (see Eq 1). Although both of them are indeed measures of variability, our results shows that their trends prior to the crashes, in some cases, can be qualitatively different (Text B and Fig N in S1 File). More specifically, although variance showed strong trends prior to all of the previous financial crashes that we analysed, volatility did now show strong and significant trends prior to some of the crashes (for example, 1987 crash in NASDAQ; 2000 crash in S&P500 and DJI). Therefore, conclusions about early warning signals based on these different measures of volatility could differ. Research on volatility has led to forecasting tools such as VIX [2]. Future research could focus on developing forecasting tools based on rising variability of detrended time series, and how it performs in comparison to those based on volatility.

As we were conducting large scale parameter scans to ensure robustness of our conclusions, we came across a couple of studies applying ideas of critical transitions and early warning signals to financial markets. In one such study, authors did not find evidence for critical slowing down prior to 2008 meltdown in the time series of USD and EUR interest rate swaps [22]. However, based on a Shannon entropic measure, they found that there is an information dissipation prior to the collapse. Our ongoing analytical and simulation work suggests that such the Shannon entropic measure is related to time series variance. Therefore, their results are broadly consistent with our analysis. However, another study claims evidence for critical slowing down prior to 2008 crash in based on housing market data [49]. It remains unclear whether those results are supported by robust parameter scans.

The aggregate market data, such as DJI, comprise of a large number of firms. Two related questions arise in the context of the relationship between macro data and its constituents: (a) Do individual constituent firms of these major markets also show similar trends? (b) What is the structure of relationship between firms, e.g. measured by cross-correlations, prior to crashes? We conducted a preliminary analysis of high frequency data of each of the 30 constituent firms of Dow Jones Index prior to 2008 crash. We found that individual firms did not show any consistent pattern of critical slowing down. However, most of the firms showed strong trends of increasing variance (Fig O in S1 File). These two patterns indicate that results of large stock markets largely hold true even at the individual firm level. In addition, the cross-correlations between most pairs of firms increased prior to the crash, consistent with a recent studies on cross-correlations [17–19, 21, 50]. These preliminary results suggest that much needs to be done in understanding how patterns of indicators at small scale firms and entities scale to large scale financial markets.

In addition to these indicators, liquidity is also argued to be one of the early warning indicators of economic crises [20, 51]. However, what would be the best measure that captures the level of liquidity in the financial markets and also that would provide a signal to the market participants about the ease of financing that exists in the market? Two most commonly used measures are: (i) the ease or availability of financing over a short term (for example, the interbank lending rates such as the London Interbank Offered Rate (LIBOR), or the LIBOR-OIS (Overnight Interest Swap) spread), (ii) the ease with which positions can be liquidated. The first measure does not directly provide an estimate of liquidity in the market; it gives an indication of the ease of credit or liquidity based on the self declared information on interest rates provided by the banks. And the second measure, based on the bid-ask spread, is the one that is most commonly used by market participants. Moreover, it has been argued that in the context of globalization of financial activity the domestic monetary aggregates may not adequately capture the ease of credit availability [20]. In any case, the analysis of LIBOR rates on their own for early warning signal for the 2008 financial crash would not be complete without a causal description of the dynamic feedback quantifying the leads and lags between the LIBOR rates or the spreads and the stock market indices. In this paper, we implicitly assumed that the market indices already incorporate the perception of about market liquidity. However, we note that quantifying the feedback between liquidity measures and the stock indices, and analyzing their dynamics within the theoretical framework of this paper would be interesting in developing early warning signals for financial crashes. We speculate that a mechanistic understanding of liquidity and stock markets [52] together with statistical models could offer development of liquidity based early warning metrics of economic boom and bursts [20, 51].

## Concluding remarks

Accurate understanding of causes of financial crashes and their prediction are both notoriously difficult owing to stochasticity inherent to these systems. Therefore, despite various limitations, developing methods to understanding nature of crashes and developing EWS of financial crashes are likely to be of wide interest for individual investors, financial industry and policy makers. Our analyses underscores the importance of stochastically driven abrupt transitions in real world scenarios, which have largely been ignored in the literature. Surprisingly, our results show that they too could be anticipated by precursors such as rising variability, although one must be cautious of false alarms. We believe that our results could have relevance and applications beyond financial markets to other complex dynamical systems that exhibit abrupt transitions.

## Methods A: Mean-field and agent based models of abrupt transitions

### A bifurcation theory based model for abrupt transitions

Consider the dynamics of a system governed by the following simple model of abrupt/catastrophic transition [53]

$$\frac{du}{dt}=-h+ru-u^{3}$$ (3)

where *u* is the state variable (stock index) with *h* and *r* as system parameters. The bifurcation diagram of (Fig 3A) is obtained by finding the equilibria (u* at which *f*(u*) = 0; equilibria are stable (unstable) if *df*/*du*|_*u* = u*_ < 0 (> 0). The state variable *u* can be in one of the two stable equilibria, which correspond to higher and lower values of the index, for an intermediate range of the parameter *h* values (Fig 3A). There exists a critical value *h*_*c*_ = 2, at which the system abruptly transits from the higher stable equilibria to the lower stable equilibria, i.e., this simple model mimics the stock market crashing from a higher value (bullish phase) to the lower values (bearish phase) of the stock index.

We include stochasticity through an additive noise term in Eq 3 and the resulting equation is given by

$$du=(-h+ru-u^{3})dt+\sigma dW$$ (4)

where *W* represents a standard Weiner process. As is well known in the literature, when the strength of stochasticity (*σ*) is small, the dynamics of the transitions described above largely remain unaffected. In contrast, when *σ* is large abrupt transitions can occur even if the system if not close to the critical points *h*_*c*_ = 2 [42, 54].

#### Early warning signals of critical transitions

Much of the focus in the literature of early warning signals of abrupt transitions is on the state variable responding discontinuously even as the parameter changes continuously (also known as critical transitions [27]). To derive early warning signals of critical transitions, the idea is to investigate the dynamics of small deviations from the stable equilibrium u* by linearizing *f*(*u*) around u*. Let *x* = *u*−u* denote the deviation from a stable equilibrium. Within the linear approximation, the Eq 4 reduces to

dx=-αxdt+σdW (5)

where *α* = |*df*/*du*|_u*_ represents the return rate to the stable equilibrium. As the system approaches the critical point *h*_*c*_, the return rate tends to zero, i.e. *α* → 0 [26, 53, 55, 56]. Furthermore, it has been shown that the statistical properties of the time series (generated by Eq 5) such as variance ($\sigma_{x}^{2}$), autocorrelation function (*C*(*τ*)) and power-spectrum (*S*(*ω*)) follow [27, 30, 32]

$$C\left(\tau \right)=\frac{1}{\alpha }\exp\left(-\alpha \tau \right)$$ (6)

$$\sigma_{x}^{2}=\frac{\sigma^{2}}{2\;\alpha }$$ (7)

$$S\left(\omega \right)=\frac{1}{2\pi }\frac{\sigma^{2}}{\alpha^{2}+\omega^{2}}$$ (8)

where *ω* represents the spectral frequency. As can be seen from the above equations, when *α* → 0 at a rate much slower than the time taken for the system to reach a steady state, the autocorrelation function and variance will both increase. Moreover, the power-spectrum will increase relatively more for smaller frequencies in comparison to higher frequencies, i.e., there will be a reddening of power-spectrum. Therefore, rising autocorrelation function (typically calculated at lag-1), variance and reddening of power-spectrum of time series data have been suggested as early warning signals of critical transitions [27, 29].

#### Early warning signals of stochastic transitions

Abrupt transitions can also occur when the parameter is far away from the critical point but the stochasticity drives the system to an alternative state. In the literature, this is referred to as stochastic transitions. However, there are various ways in which stochastic transitions can occur in a system. We describe these different routes of stochastic transitions and their generic precursors respectively in the following.

Consider the case when the parameter *h* = 0, which is relatively far away from the critical points (*h*_*c*_ = ±2) (with *r* = 3). An abrupt transition can occur when (a) a sudden large shock tips the system to the alternative state, (b) a constant but large magnitude of stochasticity (i.e., a large but constant *σ*) can lead to spontaneous switching between alternative stable states [42, 43, 54, 57], and (c) a gradually increasing strength of stochasticity (i.e., *σ* increasing with time) can also lead to switch to an alternative stable state.

For the first scenario of shock driven transitions, we clearly do not expect any early warning signals.

For the second route of large persistent stochasticity (large but constant *σ* in Eq 4) driven transitions, our numerical simulations in Figs A and B in S1 File show that consistent early warning signals do not occur (although it may be possible to find moderate/weak signals of rising variance, autocorrelation at lag-1 as well as mean power spectrum at low frequencies, for specific realizations of such a stochastic transition.)

For the third route of abrupt transition that is caused by an increasing strength of stochasticity (*σ* in Eq 4) over time, the Eqs 6, 7 and 8 suggest that whereas autocorrelation function remains unaffected, the variance and power spectrum will increase. Furthermore, since the power spectrum increases proportionately at all frequencies *ω*), there will no reddening of power spectrum. Thus, in this route to abrupt transition, we will observe no critical slowing down but an increasing trend of variance and power spectrum at all frequencies (Fig 3).

### Abrupt crashes and EWS in a heterogenous agent based behavioral economic model

Various heterogeneous [58, 59] agent based models have been developed in the field of behavioral economics [6, 43, 60–62] which have been successfully capture one or more of different *stylized facts* of markets such as volatility clustering, fat-tails in return distributions, financial meltdowns, etc. Among these models, we have chosen to simulate one of the well known heterogenous agent based models proposed by Thomas Lux [43] that captures the key phenomenon that we are interested: abrupt crashes and delayed recovery of financial markets. This model has also been shown to exhibit other stylized facts described above. Here our aim is to simulate and investigate whether there are any early warning signals (variance, autocorrelation at lag-1, reddening of power spectrum, etc) prior to abrupt crashes within this model. Thus, this analysis provides a complementary theoretical framework based on behavioral microeconomic context to investigate early warning signals and the nature of financial crashes.

#### Description of the agent based model

We describe the model developed by Thoman Lux and collaborators, (specifically: Alfarano and Lux, 2007) [44] and we follow their description of the model and parameter values as well as mathematical notations closely. The market is driven by two types of traders: Fundamentalists and Noise traders. Fundamentalists sell (or buy) fixed amount of stocks (denoted by *T*_*F*_) when the price of the stock is above a threshold value of *p*_*F*_. The number of fundamentalists, *N*_*F*_, remains constant.

Noise traders, on the other hand, could be optimists (i.e. buyers) or pessimists (i.e. sellers). They either buy or sell a fixed number, denoted by *T*_*C*_, of stock per unit time. These traders switch between their behaviors based on “herd instincts”, i.e., optimists (or pessimists) switch to become pessimists (optimists) at a rate proportional to the frequency of pessimists (optimists) among investors. Mathematically, these rates *ϕ* can be represented as

$$\phi_{O\to P}=\nu \frac{N_{P}}{N_{C}};\;\phi_{P\to O}=\frac{N_{O}}{N_{C}}$$ (9)

where *ν* is a proportionality constant. Although the number of optimists (denoted by *N*_*O*_) and pessimists (denoted by *N*_*P*_) may change since they can switch their states, the total number of noise traders, *N*_*C*_, remains constant.

The dynamics of the price *p* is given by the following equation

$$\begin{array}{ccc} \frac{dp}{dt} & = & \beta p(N_{F}T_{F}\left(p_{F}-p\right)+N_{C}T_{C}x)\\ \text{where}\;x & = & \frac{N_{O}-N_{P}}{N_{C}} \end{array}$$ (10)

The equilibrium price is given by setting *dp*/*dt* = 0 and yields

$$p=p_{F}+\frac{N_{C}T_{C}}{N_{F}T_{F}}x$$ (11)

which shows that the price may be equal to, above (or below) the fundamental value depending on the value of *x* which represents the difference in the number of optimists and pessimists.

#### Early warning signals in the behavioural economic model

Consistent with previous studies in the literature, our simulations corroborate that this heterogeneous agent based model indeed exhibits abrupt changes in the prices from a relatively high to a low value and vice versa, as seen in Fig B in S1 File. Therefore, this model captures one of the stylized facts of abrupt financial crashes and recovery.

To investigate whether there are early warning signals, we chose a window of size 1000 time series points prior to an abrupt crash in the time series generated by this model. We then computed the following indicators discussed in the previous section, namely, autocorrelation at lag-1, variance and power spectrum at low frequencies. To quantify trends in the indicators, we estimate Kendall-*τ* value. A sample of such analysis is shown in Fig B in S1 File. In this specific example, none of the indicators show increasing trends.

Since stochasticity arising from agents’ behaviours plays an important role in this model, we need to check whether these results of early warning signals are consistent across a large number of realizations. Therefore, we repeat the above calculations for 5000 realizations and plot a histogram of Kendall-*τ* values for each of the indicators (Figs A and B in S1 File). These histograms show that the indicators do not exhibit consistent increasing or decreasing trends prior to such transitions (see Fig C in S1 File for interpreting these histograms). Therefore we conclude that these indicators can not act as early warning signals of financial crashes in this behavioural economic model. Comparing this result to that shown in Fig A in S1 File, we can conclude that transitions in this agent based model is analogous to a stochastic transition generated by a simple catastrophic transition model with a large but constant strength of stochastic perturbations. This is consistent with arguments provided by Thomas Lux in his 1995 paper [43].

## Methods B: Time series analysis

### Data prior to crash

We downloaded freely available stock market data for Dow Jones Index (DJI), S&P 500 and NASDAQ. The data correspond to daily close values of all market days. We purchased historical high frequency data (one minute) from Pi Trading company. To investigate whether stock market crashes exhibited early warning signals, we selected four major crashes, namely the 1929 crash (DJI only), the 1987 crash, the 2000 crash, and the most recent 2008 crash. Well documented crash dates correspond to 24/10/1929, 19/10/1987, 10/03/2000 and 01/10/2008, respectively. To compute the indicators, we used 1000 days (roughly corresponding to four years) prior to the crash. More specifically, we first found the date in the crash year on which the stock index was maximum; for DJI these dates were 03/09/1929, 25/08/1987, 14/01/2000 and 02/05/2008, respectively and they were all at least a month before the corresponding crash dates. This method ensured that we were discarding data points too close to the crash that could potentially bias estimates of indicators. Moreover, if the indicators were to provide a signal by this date of maximum index, one can make a stronger claim about the usefulness of early warning signals. All analyses closely follow methods described in Dakos et al 2008, 2012 [28, 29]. Codes are based in open source statistical software package R and are available for download from the supplementary materials and at the Github repository (https://github.com/tee-lab/indicators-financial-meltdowns)

### Pre-processing

We removed long term trends in the chosen four year window using Gaussian kernel smoothing function, *ksmooth* in R with an input parameter *bw* representing bandwidth, and obtained residuals. All the subsequent analyses are conducted on this residual data. We then computed the indicators on a rolling window of size *l*_*rw*_. The values of parameters chosen, unless stated otherwise, are *l*_*rw*_ = 500 and *bw* = 25.

### Indicators

We estimated three recently proposed early warning signals of impending crashes, autocorrelation at lag-1, variance and power-spectrum at low frequencies, using inbuilt functions *acf*, *var* and *spectrum*, respectively. To estimate power-spectra at low frequencies, we take average of the spectrum up to 1/8^*th*^ of all frequencies (excluding the mode at zero).

We estimate the Kendall’s *τ* rank correlation, using the Kendall package, to determine whether the indicators show an increasing or a decreasing trend in the period 1 year prior to the crash. A positive (negative) value of Kendall-*τ* indicates an increasing (decreasing) trend of the indicator.

### Sensitivity analysis

While estimating Kendall-*τ*, we need to chose four parameter values: As described in the ‘pre-processing’ section above, we first chose a bandwidth to detrend the time series (*bw*) and obtain residuals. Please refer to Fig C in S1 File for a schematic illustration of three other parameter values. In that figure, the length of the window over which indicators are calculated (*l*_*rw*_) is shown using a purple arrow. Sequentially applying the chosen window (*l*_*rw*_) over the residuals, we obtain a time series of indicators. We then chose a section of this time series, highlighted in red in the same figure, of the chosen indicator for which the Kendall-*τ* is computed. Choosing this section of indicator involves two parameters, the length of the section chosen (denoted by *l*_*kw*_) and the end point of this section (denoted by *l*_*Kend*_). Unless stated otherwise, the values of the parameters are: *l*_*rw*_ = 500, *bw* = 25, *l*_*kw*_ = 250, *l*_*Kend*_ = 0.

We have done an elaborate sensitivity analysis to ensure our conclusions are not sensitive to specific choice of these parameter values. We compute trends of all three indicators for *l*_*rw*_ ranging from 375 days to 625 days at interval of 10 days (26 values), *bw* from 2.5 to 100 with an increment of 2.5 (40 values), *l*_*kw*_ from 175 to 325 days with an increment of 5 days (31 values) and *K*_*end*_ from 0 to 200 days with an increment of 5 (41 values). Thus, we have calculated Kendall’s *τ* for a total of more than 1,250,000 values.

### Estimating significance of trends (*p* values)

We ask whether the estimated Kendall-*τ* prior to the crash is typical even in years far away from crash. To do so, we have chosen a large number of time windows of length 1000 days prior to the crash being analyzed. We then computed all indicators and their Kendall-*τ* for this chosen window with the same parameter values of *l*_*rw*_, *bw*, *K*_*end*_ and *l*_*kw*_. We then computed the “p-value” which is defined as the proportion of Kendall-values in this sample that are greater than or equal to the Kendall value of the data prior to the crash. We denote this p-value by *p*_*hist*_ to represent the fact that this measure is based on comparison between a recent trend with historical trends.

We also need to test whether the observed trend is statistically significant, i.e., what is the likelihood of such Kendall-*τ* (or higher values) occurring by chance alone. The following three methods are employed to address this question.

In the first method, we generated time series that has the same Fourier spectrum and the amplitude as the original data. By original data, we mean the residuals of the detrended stock market index of length four years prior to the crash. In the second method, we fitted the original data set to an AR(1) model. We then generated time series from the fitted AR(1) model that also preserves the mean and the variance of the original data. In the third method, we bootstrap the original data by a random resampling with replacement. This method too preserves the mean and variance of the original data.

In each of the above three methods, we generated 1000 such surrogate time series and estimated the trends of indicators using Kendall-*τ*. We then calculate the corresponding p-values, denoted as *p*_*spec*_, *p*_*ar*1_ and *p*_*boot*_, defined as the proportion of Kendall-values in this sample that are greater than or equal to the Kendall value of the indicator for the stock residual data prior to the crash.

### Interpreting Kendall-*τ* with p-values

If the Kendall-*τ* is positive (negative), and if the p-value is low (high), we can conclude that the increasing (decreasing) trend of the indicator is statistically significant. We assume the standard criterion for statistical significance at 5%, which translates to a requirement of *p* < 0.05 to interpret a significant increasing trend and a *p* > 0.95 to interpret a significant decreasing trend.

Estimating p-value for each of the three methods for each parameter combination requires a large number (1000) of resampling of data. Although we did perform such sensitivity analysis (see Figs D, F and I in S1 File), this is computationally intensive. Therefore, we adopted the following strategy: First, we computed the Kendall-*τ* for the actual data based on a large parameter scan (> 1 million data points) as described in the sensitivity analysis section. We plotted a histogram of all the Kendall-*τ* for each indicator obtained by the above scan. If the histogram does show a clear peak close to 1, this implies that the trends of the corresponding indicator are not only robust to parameter variations but also that it is a strong increasing trend. In those cases, we then proceed to calculate the p-value.

However, if the histogram shows a spread-out distribution it implies that the indicators are highly sensitive to the parameter variations. Alternatively, if the histogram shows a peak at values far away from 1, it indicates a weak trend. In both of these cases, we do not proceed to compute any p-values that measures how likely the observed trend is by chance alone.

## Supporting Information

S1 File Contains all supplementary information with subsections listed below. *Text A*, Interpreting increasing variability but lack of critical slowing down prior to crashes. *Text B*, Volatility. *Fig A*, No early warning signals for stochastic transitions driven by large but constant strength of stochasticity. *Fig B*, No early warning signals for stochastic transitions in agent based models driven by large but constant strength of stochasticity. *Fig C*, Sensitivity analysis for early warning signals of crashes in DJI. *Fig D*, Sensitivity of p-values for all three indicators for all crashes in DJI. *Fig E*, Early warning signals of major financial crashes of S&P 500 for three major crashes of 1987, 2000 and 2008. *Fig F*, Sensitivity analysis for all three indicators for all crashes in S&P 500. *Fig G*, Recent trends of EWS for S&P 500. *Fig H*, Early warning signals of major financial crashes of NASDAQ for three major crashes of 1987, 2000 and 2008. *Fig I*, Sensitivity analysis for the results of NASDAQ. *Fig J*, Recent trends of EWS for NASDAQ. *Fig K*, Onset of strong trends of indicators roughly coincide across three stock markets. *Fig L*, Early warning signals of financial crashes of German and UK stock indices in 2000 and 2008. *Fig M*, Recent trends of German (DAX) and UK (FTSE) stock market indices. *Fig N*, Results of volatility. *Fig O*, Early warning signals of financial crashes for individual firms of DJI in 2008 and Cross-correlations.
(PDF)
