# Supplementary material for: Lack of Critical Slowing Down Suggests that Financial Meltdowns Are Not Critical Transitions, yet Rising Variability Could Signal Systemic Risk
Source: PLoS One. 2016 Jan 13;11(1):e0144198. doi: 10.1371/journal.pone.0144198 (PMC4711996; doi:10.1371/journal.pone.0144198)
Supplement: S1 File — Text A, Interpreting increasing variability but lack of critical slowing down prior to crashes. Text B, Volatility. Fig A, No early warning signals for stochastic transitions driven by large but constant strength of stochasticity. Fig B, No early warning signals for stochastic transitions in agent based models driven by large but constant strength of stochasticity. Fig C, Sensitivity analysis for early warning signals of crashes in DJI. Fig D, Sensitivity of p-values for all three indicators for all crashes in DJI. Fig E, Early warning signals of major financial crashes of S&P 500 for three major crashes of 1987, 2000 and 2008. Fig F, Sensitivity analysis for all three indicators for all crashes in S&P 500. Fig G, Recent trends of EWS for S&P 500. Fig H, Early warning signals of major financial crashes of NASDAQ for three major crashes of 1987, 2000 and 2008. Fig I, Sensitivity analysis for the results of NASDAQ. Fig J, Recent trends of EWS for NASDAQ. Fig K, Onset of strong trends of indicators roughly coincide across three stock markets. Fig L, Early warning signals of financial crashes of German and UK stock indices in 2000 and 2008. Fig M, Recent trends of German (DAX) and UK (FTSE) stock market indices. Fig N, Results of volatility. Fig O, Early warning signals of financial crashes for individual firms of DJI in 2008 and Cross-correlations. (PDF) [file pone.0144198.s001.pdf]

Supporting Information: Lack of critical slowing down suggests that financial meltdowns are not critical transitions, yet rising variability signals systemic risk

Vishwesha Guttal*, Srinivas Raghavendra, Nikunj Goel and Quentin Hoarau
*Address for correspondence: guttal@ces.iisc.ernet.in (VG)

List of Figures

A No early warning signals for stochastic transitions driven by large but constant strength of stochasticity. 4
B No early warning signals for stochastic transitions in agent based models driven by large but constant strength of stochasticity. 5
C Sensitivity analysis for early warning signals of crashes in DJI. 6
D Sensitivity of p-values for all three indicators for all crashes in DJI. 7
E Early warning signals of major financial crashes of S&P 500 for three major crashes of 1987, 2000 and 2008. 8
F Sensitivity analysis for all three indicators for all crashes in S&P 500. . . 9
G Recent trends of EWS for S&P 500 10
H Early warning signals of major financial crashes of NASDAQ for three major crashes of 1987, 2000 and 2008. 11
I Sensitivity analysis for the results of NASDAQ 12
J Recent trends of EWS for NASDAQ 13
K Onset of strong trends of indicators roughly coincide across three stock markets 14
L Early warning signals o financial crashes of German and UK stock indices in 2000 and 2008. 15
M Recent trends of German (DAX) and UK (FTSE) stock market indices . . 16
N Results of volatility 18
O Early warning signals of financial crashes for individual firms of DJI in 2008 and Cross-correlations. 19

Text A: Interpreting increasing variability but lack of critical slowing down prior to crashes

Our analysis of financial markets prior to crashes show lack of critical slowing down, lack of reddening of power spectra but increasing variability and increase in power spectrum at all frequencies (Fig 1 of main text; Figures SE and SH). How do we interpret these puzzling results?

When an increasing strength of noise leads to the stochastic transition, we find from Eqs 4, 5 and 6 of the main text that the autocorrelation function will remain unaffected but the time series variance and spectrum both increase (Fig 3 of main text). However, note that power-spectrum (Eq 6) at any frequency is affected by α (a measure of proximity to the critical point) and σ (strength of external perturbations). An increasing strength of perturbations will lead to increasing power spectrum at all frequencies. Contrast this with the case of how power-spectrum of the same Eq (6) increases for the case of critical slowing down, i.e. $\alpha \to 0$; here, although the power-spectrum again increases for all frequencies, the impact is more for lower-frequencies leading to amplification of strength of the spectrum at lower-frequencies in comparison to higher frequencies.

In other words, increasing autocorrelation at lag-1 and reddening of power spectrum could be a consequence of critical slowing down but not due to increasing strength of perturbations. When the transition is caused by an increasing strength of stochastic perturbations, we expect to see increasing variance but not autocorrelation or reddening of power spectrum. Conversely, if we were to observe an abrupt transition that is preceded by an increase in both variance and power spectrum (but no reddening) but with no significant trends in autocorrelation at lag 1, we may conclude that the transition is not preceded by critical slowing down. Rather, it is more likely driven by an increasing strength of stochastic perturbations.

Text B: Volatility

Definition

Volatility has been studied extensively as a statistical measure of uncertainty in finance markets. It has also been proposed as an early warning signal of financial crashes. Here, we comment on whether volatility provides a consistent early warning signal in real data.

Although there are various definitions of volatility that slightly differ from each other, here we employ the definition of Poon and Granger[1]. Volatility can be defined as a standard deviation, σ, or variance σ^2, of stock returns

$$\hat{\sigma}^2 = \sum_{t=1}^{T} \frac{(R_t - \bar{R})^2}{T-1} \tag{1}$$

where $\bar{R}$ is the mean of the returns. The return itself is calculated from the stock prices p_t as

$$R_t = \log\left[\frac{p_{t+1}}{p_t}\right] \approx \frac{p_{t+1} - p_t}{p_t} \tag{2}$$

The approximated formulate above is reasonable for analysis of high frequency data.

Volatility in DJI, SP500 and NASDAQ data prior to crashes

Here, we have computed volatility and its trends for each of the crashes using Dow Jones Index (DJI), S&P 500 and NASDAQ indices. For the Dow Jones Index, we find that volatility does increase prior to financial crashes of 1929, 1987 and 2008 but not for the 2000 crash. For the SP500 index, the volatility again increases prior to 1987 and 2008 crashes, but not for the 2000 crash. In contrast, for the NASDAQ index, the volatility shows no signatures prior to 1987 crash but it does show relatively strong trends prior to both 2000 and 2008 crashes.

We summarize the results as follows. First, volatility did increase prior to various financial crashes, but there are exceptions and some crashes were not preceded by any clear trends. Second, it may be argued that variance of residuals that we calculated and showed in the main text is just another measure of volatility. We recall that variance showed clear increasing trends prior to all the financial crashes that we analyzed. However, the lack of clear trends in volatility prior to some of the crashes show that variance and volatility may lead to different conclusions about impending financial crashes.

We further note that the the numerator of the return rate is difference between consecutive price indices. If the mean of the stock index does not change much over time scale T over which volatility is calculated, then p_t is nearly a constant and the return rate can be interpreted as the scaled detrended time series. In other words, R_t would be scaled residuals, and therefore volatility would be same as variance within a scaling factor. However, as these results show, this approximation is not always true since results of variance and volatility can be qualitative and quantitative different.

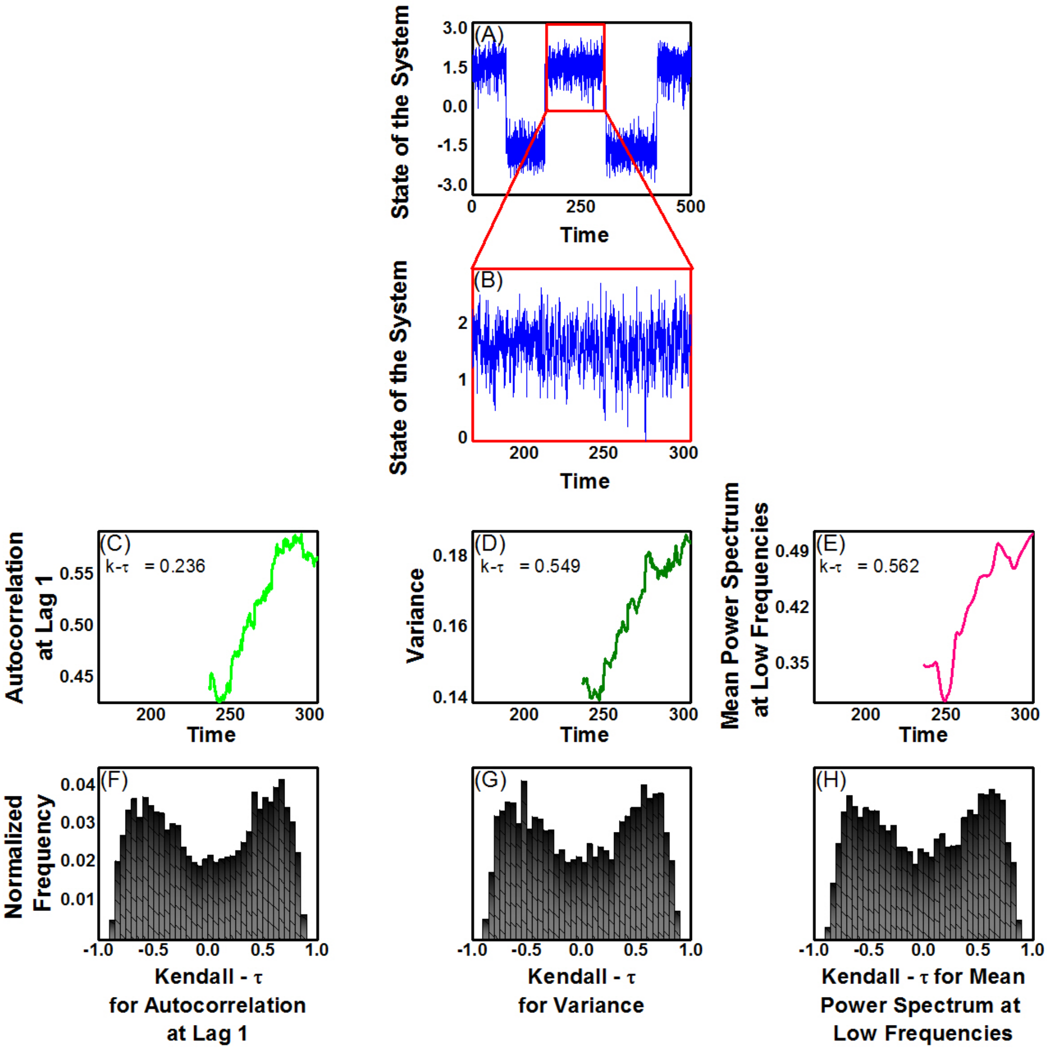

Figure A: No early warning signals for stochastic transitions driven by large but constant strength of stochasticity. (A) shows a representative time series of stochastic transitions where the strength of the external perturbation was large but kept constant. We obtained this time series by simulating Eq 2 of the main text with $h = 0, r = 3, \sigma = 1.1$ and time step of integration $dt = 0.01$. (B) shows the window of time series prior to a transition. For this window of time series, (C), (D), and (E) show trends of indicators acf at lag-1, variance and power-spectrum at low frequencies, respectively. The Kendall-τ mentioned within each these panels indicate weak/moderate trends prior to transitions. The next row, (F), (G) and (H) show histogram of Kendall-τ for each of those indicators based on 10000 such time series windows prior to transitions. These histograms show that the indicators do not exhibit consistent increasing or decreasing trends prior to such transitions.

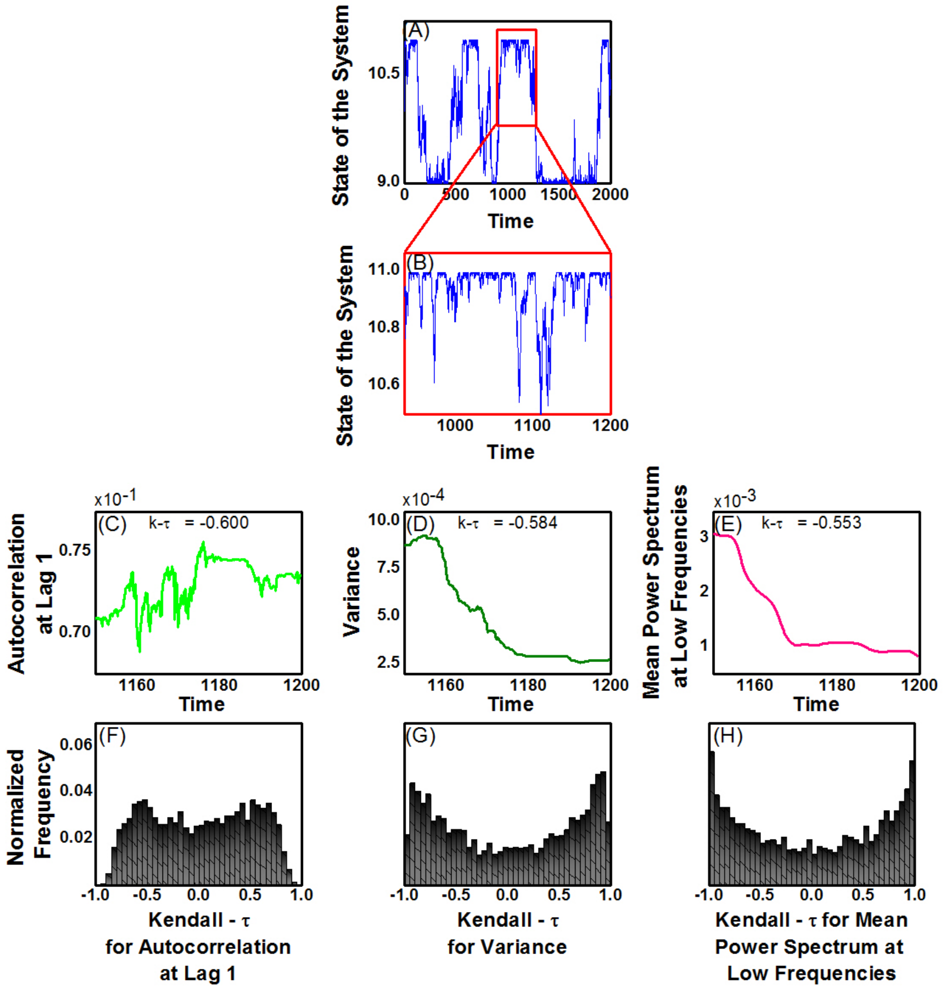

Figure B: No early warning signals for stochastic transitions in agent based models driven by large but constant strength of stochasticity. Time series and lack of early warning signals of abrupt financial crashes in the behavioral economic model of Alfarano and Lux, 2007[2]. The model parameters we used are: $N_C = N_F = 100, \nu = 0.05, l_{rw} = 500, bw = 25$.

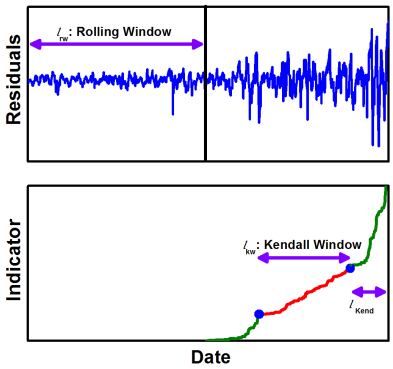

(a)

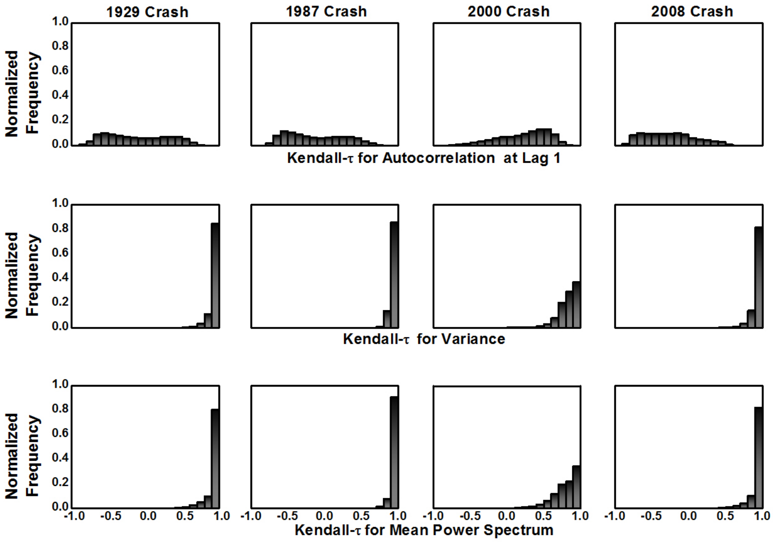

(b)

Figure C: Sensitivity analysis for early warning signals of crashes in DJI. (a) A schematic representation of three key parameters that are chosen to compute indicators and their trends. See 'Sensitivity analysis' under Methods B for explanation of the figure. (b) **Sensitivity analysis for early warning signals of DJI:** For the Dow Jones Index, sensitivity of Kendall-τ of acf at lag-1 (shown in top row), variance (middle row) and power spectrum (bottom row) to choice of parameter values (over 1 million parameters; see Sensitivity analysis under Methods B). It shows that the trends of acf at lag-1, whose histogram is nearly flat in the entire domain of $[-1, 1]$, is highly sensitive to the choice of parameters and this is true for all crashes. In contrast, the histograms of Kendall-τ for variance and power spectrum peak with a narrow width at values of Kendall-τ close to 1 implying strongly increasing trends.

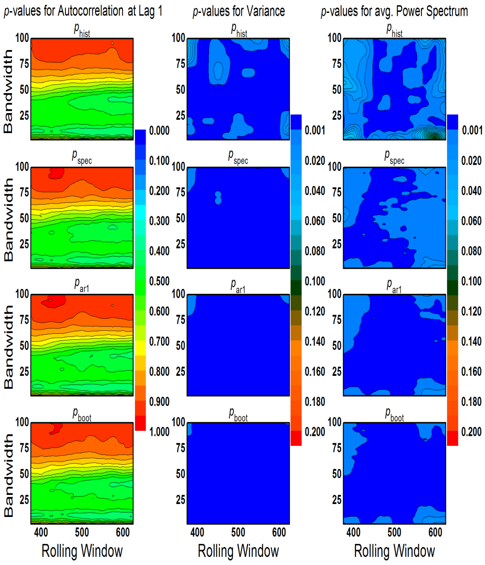

(a) Sensitivity of p-values for 1929 crash

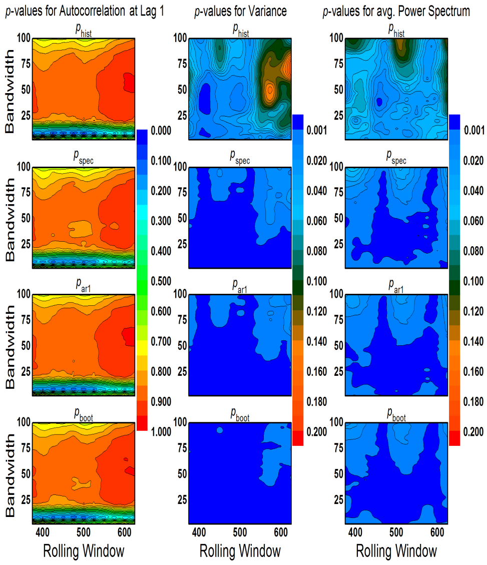

(b) Sensitivity of p-values for 1987 crash

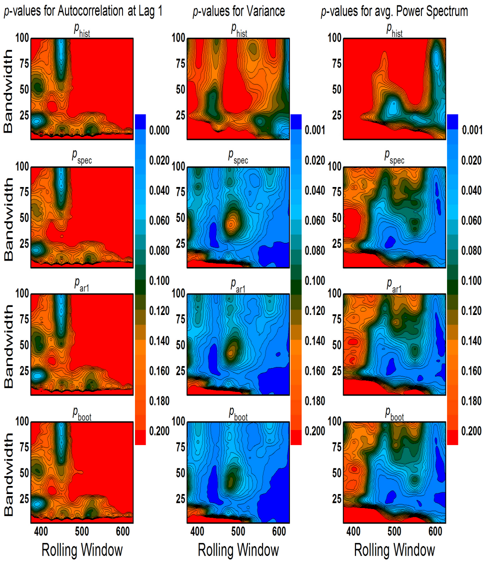

(c) Sensitivity of p-values for 2000 crash

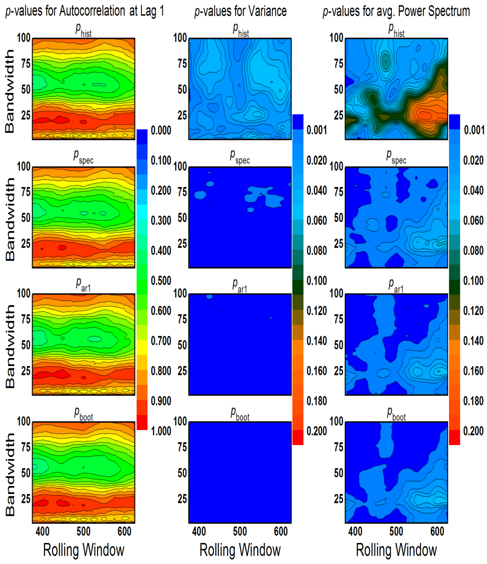

(d) Sensitivity of p-values for 2008 crash

Figure D: Sensitivity of p-values for all three indicators for all crashes in DJI. We clearly find that the p-values for trends of variance and average power spectrum are fairly robust and relatively small (< 0.001 for most cases) whereas that of acf at lag 1 is large and sensitive to parameter values. Therefore, our conclusion that there is no evidence for critical slowing down, but a strong trend of increasing variance & power spectrum occurred prior to crashes is robust. A mild exception is that of 2000 crash where statistical support for all indicators were somewhat weak in comparison to other crashes (See Methods B). In addition, except for 2000 crash, the scale for plots of acf at lag 1 is much large (0 to 1) in comparison to those of variance and power spectrum which is much smaller (0 to 0.2).

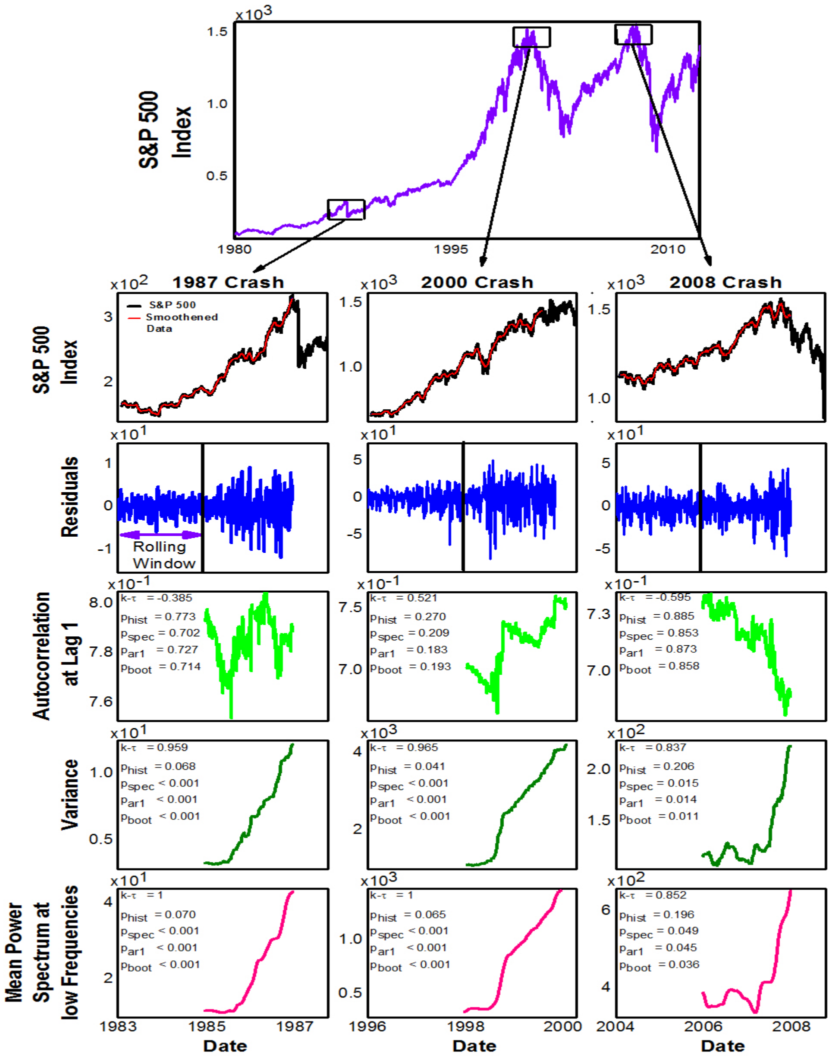

Figure E: Early warning signals of major financial crashes of S&P 500 for three major crashes of 1987, 2000 and 2008. All methods and parameters were as in Fig 1 of the main text but for the data of S&P 500 stock index. As in the case of DJI, autocorrelation at lag 1 does not show any clear trends, suggesting no evidence for critical slowing down prior to any of these crashes in S&P 500. Furthermore, a strong trend of increasing variance and average power spectrum precedes all the three transitions. Parameters: $l_{rw} = 500, bw = 25, l_{kw} = 250, l_{Kend} = 0$ (See Methods B)

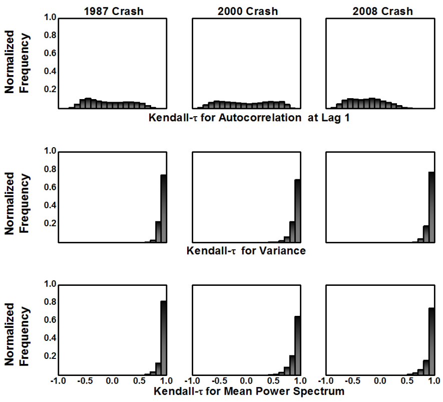

(a) Sensitivity of Kendall-τ for S&P 500

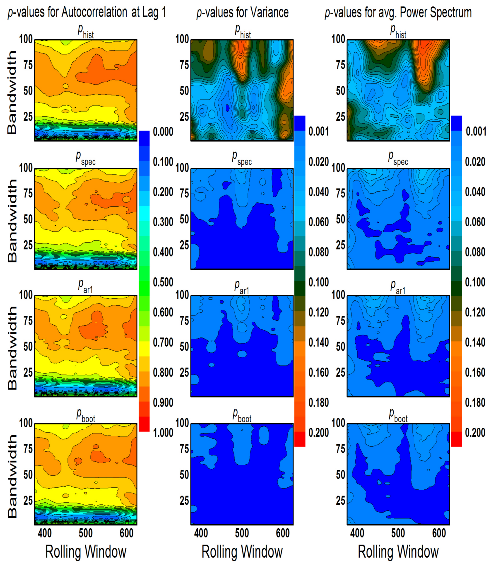

(b) Sensitivity of p-values for 1987 crash

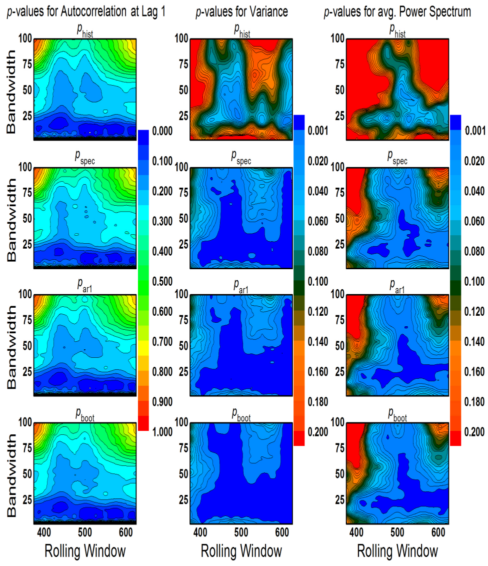

(c) Sensitivity of p-values for 2000 crash

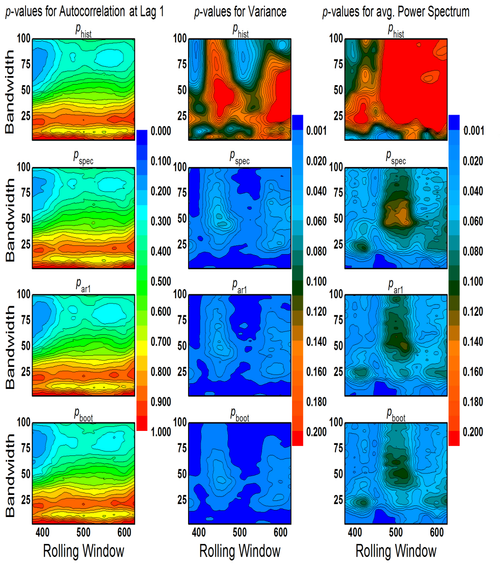

(d) Sensitivity of p-values for 2008 crash

Figure F: Sensitivity analysis for all three indicators for all crashes in S&P 500. (a) The trends in acf at lag-1 were sensitive to variations in parameters whereas those of variance and power spectrum were robust (see Sensitivity analysis under Methods B) (b-d) We clearly find that the p-values for trends of variance and average power spectrum are fairly robust and relatively small (< 0.001 for most cases) whereas that of acf at lag 1 is large and sensitive to parameter values. Therefore, our conclusion that there is no evidence for critical slowing down, but a strong trend of increasing variance & power spectrum occurred prior to crashes is robust (See Methods B). Note that the scale for plots of acf at lag 1 is much large (0 to 1) in comparison to those of variance and power spectrum which is much smaller (0 to 0.2).

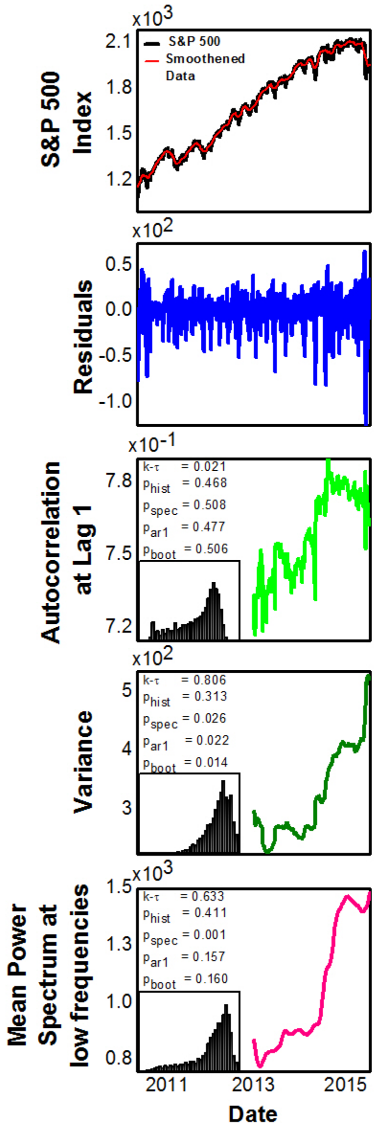

Figure G: Recent trends of EWS for S&P 500. Lack of strong increasing trends of indicators for S&P 500 data from 19/09/2011 to 18/09/2015 suggest there may be no imminent financial crash. Parameters used to analyse data are same as in Fig 1 ($l_{rw} = 500, bw = 25, l_{kw} = 250, l_{Kend} = 0$). The insets show a distribution of Kendall-τ for the corresponding indicator obtained by roughly 1.25 million parameter combinations (see Sensitivity analysis under Methods B). Lack of consistent value in $k - \tau$ and relatively high p-values (> 0.1) for all EWS suggest that there are no clear trends. The scale on x-axis is from -1 to 1 whereas on the y-axis is from 0 to 0.12.

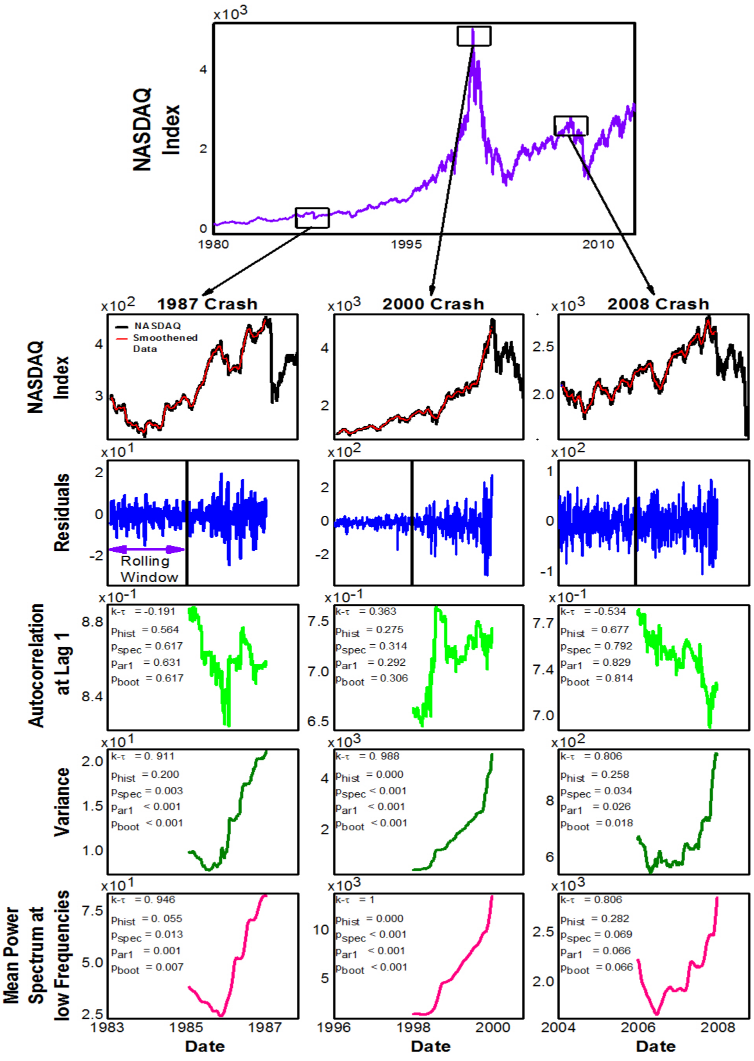

Figure H: Early warning signals of major financial crashes of NASDAQ for three major crashes of 1987, 2000 and 2008. All methods and parameters were as in Fig 1 of the main text but for the data of NASDAQ stock index (Parameters: $l_{rw} = 500, bw = 25, l_{kw} = 250, l_{Kend} = 0$). As in results of DJI, autocorrelation at lag 1 does not show any clear trends, suggesting no evidence for critical slowing down prior to any of these crashes in NASDAQ. Furthermore, a strong trend of increasing variance and average power spectrum precedes all the three transitions (See Methods B).

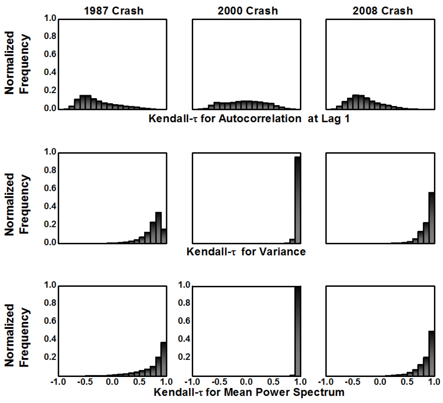

(a) Sensitivity of Kendall-τ for NASDAQ

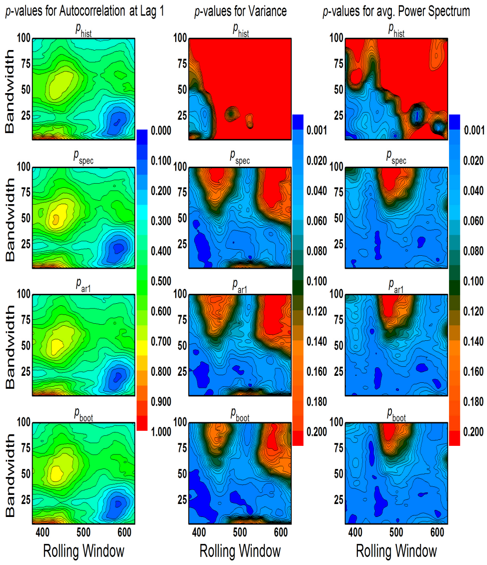

(b) Sensitivity of p-values for 1987 crash

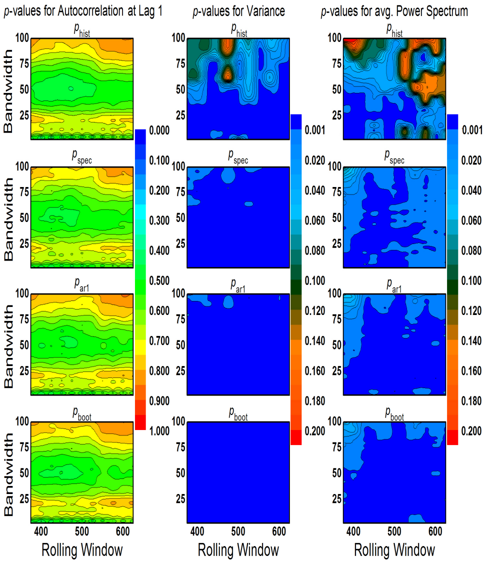

(c) Sensitivity of p-values for 2000 crash

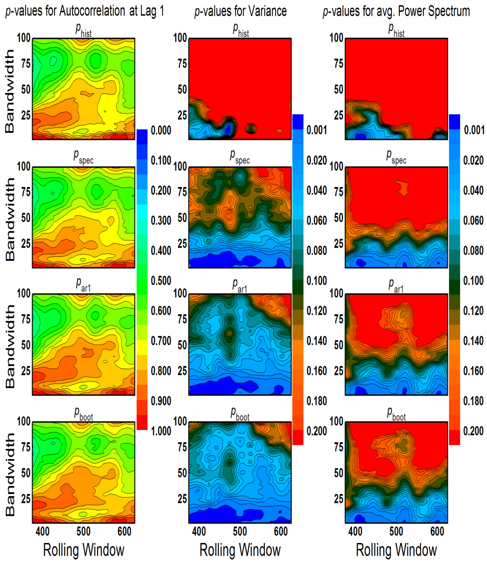

(d) Sensitivity of p-values for 2008 crash

Figure I: Sensitivity analysis for the results of NASDAQ. (a) The trends of acf at lag-1 is sensitive to parameter variations whereas those of variance and power spectrum are not (see Sensitivity analysis under Methods B). (b-d) We clearly find that the p-values for trends of variance and average power spectrum are fairly robust and relatively small (< 0.001 for most cases) whereas that of acf at lag 1 is large and sensitive to parameter values. Therefore, our conclusion that there is no evidence for critical slowing down, but a strong trend of increasing variance & power spectrum occurred prior to crashes is robust (See Methods B). However, in comparison to the results of DJI, the overall statistical support is weaker. An exception is that of 2000 crash: Strong trends prior to 2000 crashes are broadly consistent with the fact that 2000 financial crash, also called Dot com bubble, was caused by crises in the Information Technology sector that directly affects NASDAQ. Note that the scale for plots of acf at lag 1 is much large (0 to 1) in comparison to those of variance and power spectrum which is much smaller (0 to 0.2).

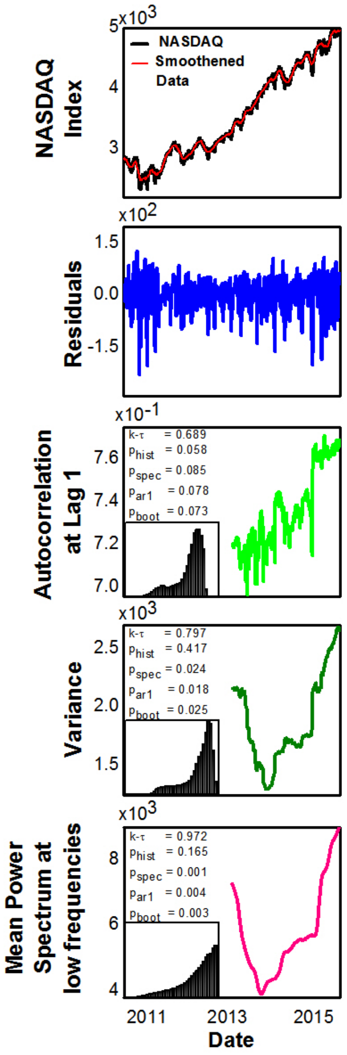

Figure J: Recent trends of EWS for NASDAQ. Strong increasing trends of variance and power-spectrum for NASDAQ data from 19/09/2011 to 18/09/2015 suggest we may be headed toward a potential financial crisis in NASDAQ. Parameters: $l_{rw} = 500, bw = 25, l_{kw} = 250, l_{Kend} = 0$). Note that, although the specific plot of trends of power spectrum at low frequencies shows a strong trend (with Kendall-$\tau > 0.9$), the trend is somewhat sensitive (although with a peak close to 1, suggesting strong increasing trends are found for a larger number of parameter values) to the choice of parameter values as shown by the inset showing the distribution of Kendall-τ based on parameter scan. The insets show a distribution of Kendall-τ for the corresponding indicator obtained by roughly 1.25 million parameter combinations (see Sensitivity analysis under Methods B). The scale on x-axis is from -1 to 1 whereas on the y-axis is from 0 to 0.2.

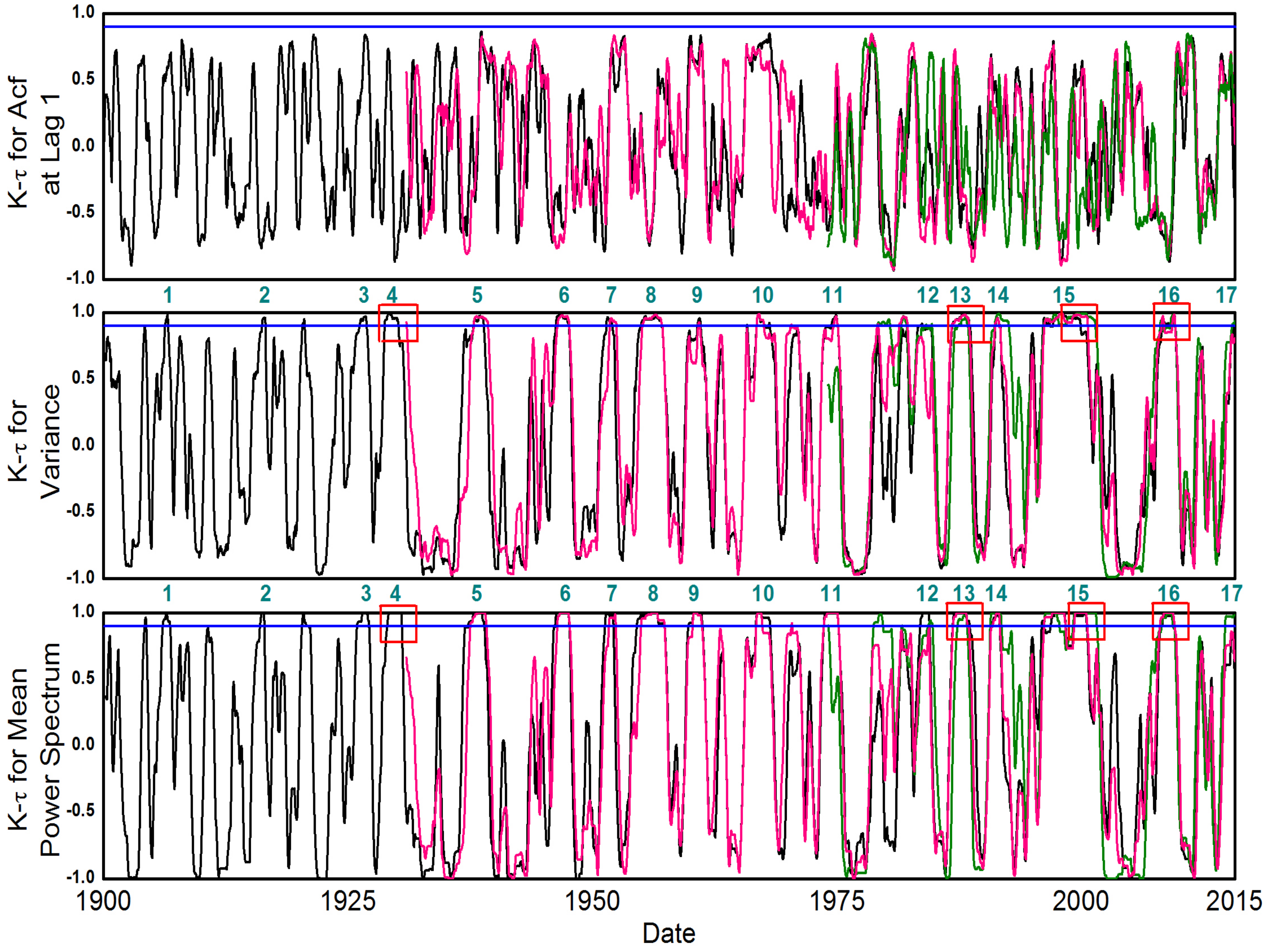

Figure K: Onset of strong trends of indicators roughly coincide across three stock markets. Here, we have shown the temporal trend of strength of indicators, as in Fig 2 of main text, in the same plot for DJI (black line), S&P 500 (magenta) and NASDAQ (green). We observe that the onset of strong increasing trend of indicators, i.e. when the corresponding Kendall-τ exceeds a value of 0.9, roughly match for all three markets.

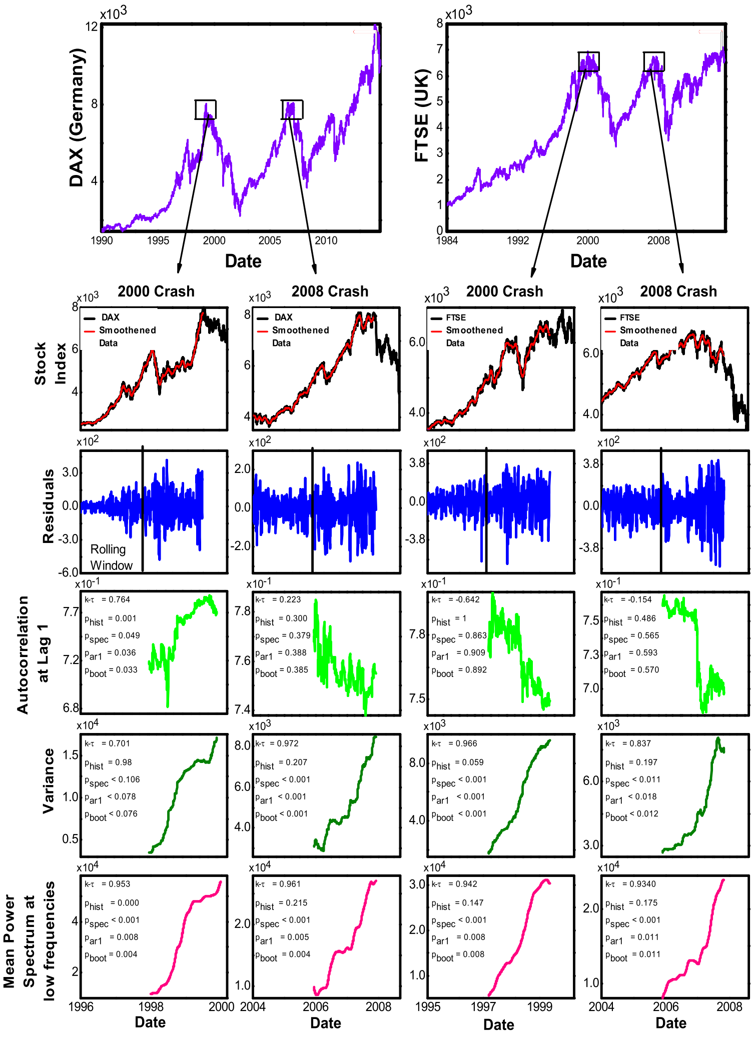

Figure L: Early warning signals o financial crashes of German and UK stock indices in 2000 and 2008. All methods and parameters were as in Fig 1 of the main text but for the data of DAX (German) and FTSE (UK) stock index (Parameters: $l_{rw} = 500, bw = 25, l_{kw} = 250, l_{Kend} = 0$). As in results of DJI, autocorrelation at lag 1 does not show any clear trends, suggesting no evidence for critical slowing down prior to any of these crashes in either of these two markers. Furthermore, a strong trend of increasing variance and average power spectrum precedes all the three transitions (See Methods B).

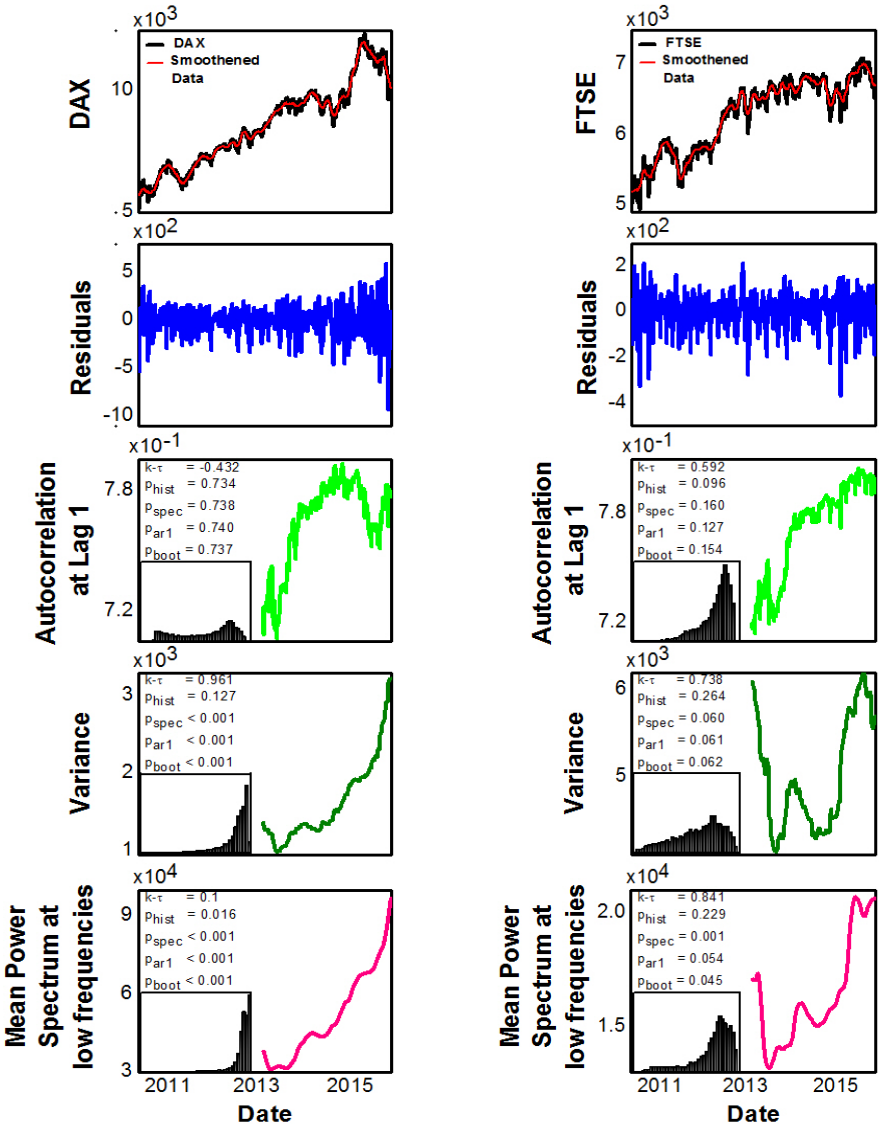

(a) German Index

(b) UK Index

Figure M: Recent trends of German (DAX) and UK (FTSE) stock market indices. Strong increasing trends of variance and power-spectrum for DAX but not for the FTSE stock market data from 19/09/2011 to 18/09/2015 (Parameters: $l_{rw} = 500, bw = 25, l_{kw} = 250, l_{Kend} = 0$). The insets show a distribution of Kendall-τ for the corresponding indicator obtained by roughly 1.25 million parameter combinations (see Sensitivity analysis under Methods B). Note that the trend is not sensitive to parameter values for DAX (look at the inset where the peak of histogram of Kendall-τ is close to 1) whereas it is sensitive for FTSE. The scale on x-axis is from -1 to 1. The y-axis is from 0 to 0.12 for DAX and from 0 to 0.25 for DAX.

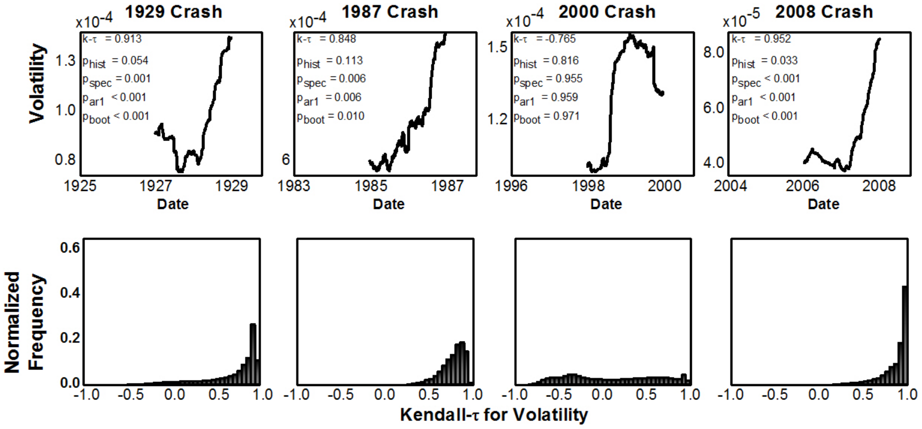

(a) Trends of volatility in DJI

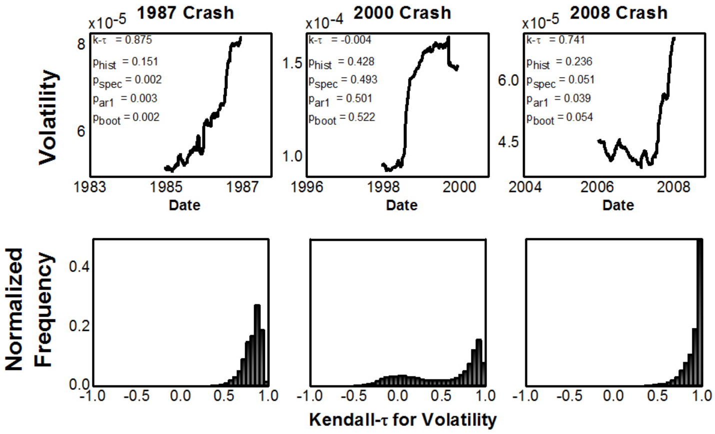

(b) Trends of volatility in S&P 500

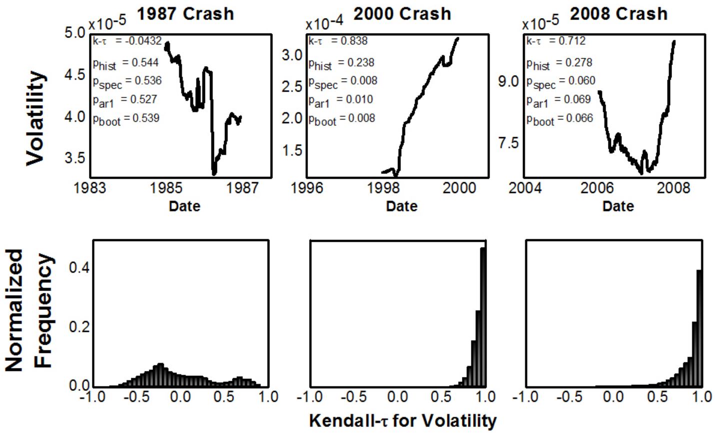

(c) Trends of volatility in NASDAQ

Figure N: Results of volatility. First row shows trends of volatility prior to the financial crashes in different stock markets with parameter values used of $l_{rw} = 500$, $bw = 25$, $l_{kw} = 250$ and $K_{end} = 0$. The histograms in the second row show histogram of the Kendall-τ calculated based on about 1.25 million parameter value combinations (See Methods B). Therefore, this can be used to interpret how sensitive (and consistent) are trends to our choice of parameter values.

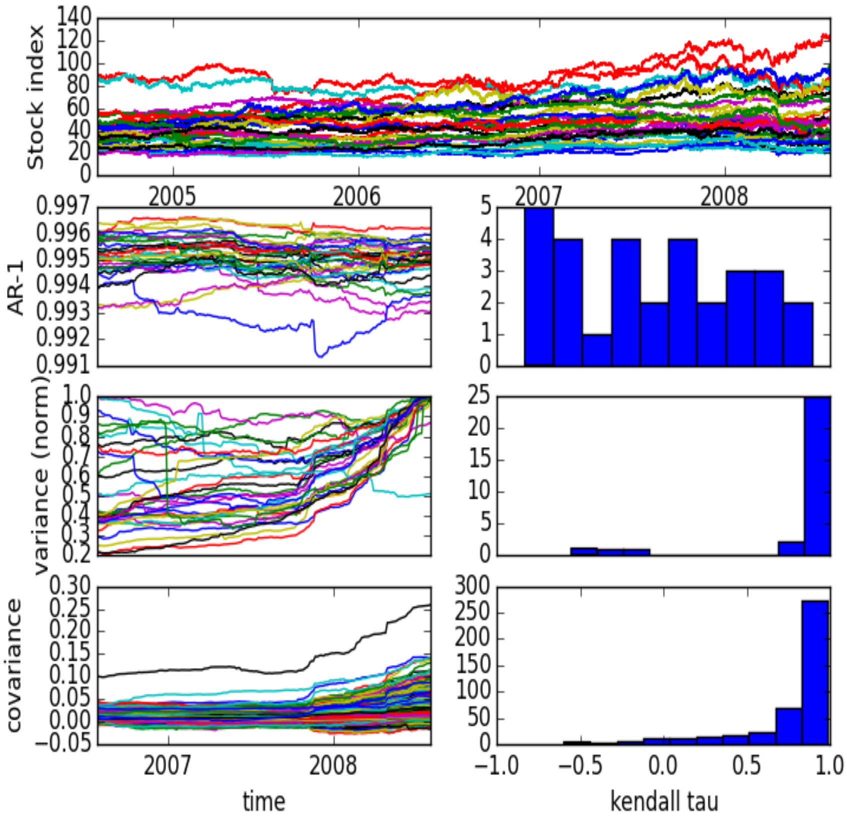

Figure O: Early warning signals of financial crashes for individual firms of DJI in 2008 and Cross-correlations. All methods and parameters were as in Fig 1 of the main text but for the data of individual firms of DJI. We employed a high frequency minute data for this analysis (the frequency may differ slightly between firms). The first row shows time series of all individual firm data. Second row shows the trends of autocorrelations for each of the firms (left column) and a histogram of strengths of trends (right column). The histogram shows that there was no consistency in the behaviour of autocorrelation at lag-1 across firms. Therefore, we may conclude that lack of critical slowing down extends down at firm level as well. The third row shows trends of variance and histogram of strengths for all firms. Clearly, most firms are showing a strong increasing trend, which is consistent with the result at the entire market level as well. Fourth row shows covariance between all pairs of firms and the corresponding Kendall-tau histogram suggests an increasing for most firm-pairs. Future work can investigate these results in more depth.

References

[1] Poon SH, Granger CW (2003) Forecasting volatility in financial markets: A review. *Journal of economic literature* 41(2):478–539.

[2] Alfarano S, Lux T (2007) A minimal noise trader model with realistic time series properties. pp. 345–361.
